# Metabolic Characterization of a Rare Genetic Variation Within *APOC3* and Its Lipoprotein Lipase–Independent Effects

**DOI:** 10.1161/CIRCGENETICS.115.001302

**Published:** 2016-06-21

**Authors:** Fotios Drenos, George Davey Smith, Mika Ala-Korpela, Johannes Kettunen, Peter Würtz, Pasi Soininen, Antti J. Kangas, Caroline Dale, Debbie A. Lawlor, Tom R. Gaunt, Juan-Pablo Casas, Nicholas J. Timpson

**Affiliations:** From the MRC Integrative Epidemiology Unit, School of Social and Community Medicine, University of Bristol, Bristol (F.D., G.D.S., M.A.-K., D.A.L., T.R.G., N.J.T.); Institute of Cardiovascular Science, University College London, London, United Kingdom (F.D., C.D., J.-P.C.); Computational Medicine, Faculty of Medicine, University of Oulu & Biocenter Oulu, Oulu (M.A.-K., J.K., P.W., P.S., A.J.K.); NMR Metabolomics Laboratory, School of Pharmacy, University of Eastern Finland, Kuopio (M.A.-K., J.K., P.S.); Public Health Genomics Unit, Department of Chronic Disease Prevention, National Institute for Health and Welfare, Helsinki, Finland (J.K.); and Department of Non-communicable Disease Epidemiology, London School of Hygiene and Tropical Medicine, London, United Kingdom (C.D., J.-P.C.).

**Keywords:** association studies, genetics, lipids, LPL, metabolism, triglycerides, VLDL

## Abstract

**Background—:**

Plasma triglyceride levels have been implicated in atherosclerosis and coronary heart disease. Apolipoprotein C-III (APOC3) plays a key role in the hydrolysis of triglyceride-rich lipoproteins to remnant particles by lipoprotein lipase (LPL) and their uptake by the liver. A rare variant in *APOC3*(rs138326449) has been associated with triglyceride, very low–density lipoprotein, and high-density lipoprotein levels, as well as risk of coronary heart disease. We aimed to characterize the impact of this locus across a broad set of mainly lipids-focused metabolic measures.

**Methods and Results—:**

A high-throughput serum nuclear magnetic resonance metabolomics platform was used to quantify 225 metabolic measures in 13 285 participants from 2 European population cohorts. We analyzed the effect of the *APOC3* variant on the metabolic measures and used the common *LPL*(rs12678919) polymorphism to test for LPL-independent effects. Eighty-one metabolic measures showed evidence of association with *APOC3*(rs138326449). In addition to previously reported triglyceride and high-density lipoprotein associations, the variant was also associated with very low–density lipoprotein and high-density lipoprotein composition measures, other cholesterol measures, and fatty acids. Comparison of the *APOC3* and *LPL* associations revealed that *APOC3* association results for medium and very large very low–density lipoprotein composition are unlikely to be solely predictable by the action of APOC3 through LPL.

**Conclusions—:**

We characterized the effects of the rare *APOC3*(rs138326449) loss of function mutation in lipoprotein metabolism, as well as the effects of *LPL*(rs12678919). Our results improve our understanding of the role of *APOC3* in triglyceride metabolism, its *LPL* independent action, and the complex and correlated nature of human metabolites.

## Abstract 2

Supplemental Digital Content is available in the text.

High triglycerides levels have been consistently linked to risk of cardiovascular disease,^1^ with mounting evidence supporting their causal role in the progression of the disease.^2–5^ In moderately raised concentrations (2–10 mml/L),^6^ triglycerides appear to be able to penetrate the arterial intima^7^ where they are trapped within the arterial wall contributing to atherosclerosis.^8^ In the fasting state, circulating triglycerides are transported in large, medium, and small particles of very low–density lipoproteins (VLDLs) and their remnants after lipolysis and remodeling,^9^ mainly in the form of small VLDLs and intermediate-density lipoproteins. Smaller percentages of triglyceride can also be found in low-density lipoprotein (LDL; <10%) and high-density lipoprotein (HDL; ≈15%) particles.^10^ In postprandial conditions, chylomicrons and their remnants account for a large proportion of the elevated triglyceride levels.^11^

**Clinical Perspective on p 239**

A common component of triglyceride-rich lipoproteins is apolipoprotein C-III (APOC3). APOC3 is a small 99 amino acid peptide^12^ coded by the *APOC3* gene located on chromosome 11, between and in close proximity to the *APOA4* and *APOA1* genes.^13^ Recent advances in genetic data collection have permitted the study of low minor allele frequency variants (<5% minor allele frequencies) which have, potentially, strong associations with phenotypes. Through these methods, a rare loss of function single nucleotide variant rs138326449 that changes the splicing of the *APOC3* gene^10,14–16^ has been identified. The rare allele of this single nucleotide variant has been associated with a substantial decrease in the risk of coronary artery disease,^10,15^ varying levels of reduction in triglyceride of 0.5 to 1.5 mmol/L depending on the population studied and evidence of changes to VLDL and HDL levels.^16^

APOC3 is involved in several intra- and extracellular mechanisms, including the production and clearance of triglyceride-rich lipoproteins from circulation. The effect of APOC3 on triglyceride and remnant particles, smaller and denser remodeled triglyceride-rich particles with some of their triglyceride removed, is suggested to operate mainly through the inhibition of triglyceride-rich lipoproteins hydrolysis by lipoprotein lipase (LPL)^17^ and a subsequent attenuation of uptake into hepatocytes.^12^ *LPL* polymorphisms have been associated with levels of both triglyceride and HDL^18^ and also with risk of coronary artery disease.^19^ To date, epidemiological studies have not had the required information to assess the molecular mechanisms involved, and despite the consensus that APOC3 is affecting the TLRs, small in vivo and in vitro studies have not determined the relative importance of the different mechanisms^20^ and how specific mutations of the *APOC3* gene can affect these processes.^21^

Here we use data from 2 well-characterized European population cohort studies to provide a detailed profile of the associations between the rare and poorly characterized *APOC3* genetic variant rs138326449 and individual lipoprotein subclasses that might contribute to atherosclerosis risk. For this, we used a targeted metabolomics approach measuring, among others, the size and composition of 14 lipoprotein subclasses. This approach has previously been used to characterize the molecular profile of common diseases, identify new biomarkers, and study the genetic basis of systemic metabolism (reviewed in Soininen et al^22^). We aimed to characterize in greater detail the impact of variation at this locus and its role in triglyceride metabolism as seen from an epidemiological perspective, including elucidating APOC3’s LPL-dependent and LPL-independent actions on the levels and composition of specific lipoprotein particles, as well as the mechanism of action of a recently proposed *APOC3* inhibitor for the treatment of hypertriglyceridemia.

## Methods

### Study Populations

The Avon Longitudinal Study of Parents and Children (ALSPAC) is a population-based, prospective birth cohort (www.bris.ac.uk/alspac). The study initially invited >14 000 pregnancies and has since followed participants in several phases during development and maturity. Information on the phases can be found at www.bris.ac.uk/alspac/researchers/resources-available/data-details/data-tables/documents/focusclinicsessions.pdf. Full details of the study have been published previously, and here focus is on the offspring of this study (herein referred to as young participants) and their mothers.^23^ Ethical approval for the study was obtained from the ALSPAC Ethics and Law Committee and from the UK National Health Service Local Research Ethics Committees. Participants have provided informed consent for the use of the data.

Analyses were also undertaken in an independent cohort of women. The British Women Heart Health Study (BWHHS) is a prospective cohort study that recruited women between the ages of 60 and 79 years from 23 towns across the United Kingdom between 1999 and 2001 and has followed those women forward through record linkage and detailed questionnaires since that time.^24^ Ethical approval for the study was obtained from the UK National Health Service Research Ethics Committees, and participants provided informed consent.

### Serum Nuclear Magnetic Resonance Metabolomics

A high-throughput serum nuclear magnetic resonance metabolomics platform was used to quantify ≤233 metabolic measures that represent a broad molecular signature of systemic metabolism.^22,25^ The measured set covers multiple metabolic pathways, including lipoprotein lipids and subclasses, fatty acids and fatty acid compositions, as well as amino acids and glycolysis precursors. All molecular measures are quantified in a single experimental setup, constituting both established and novel metabolic risk factors.^22^ The applied nuclear magnetic resonance–based metabolic profiling has recently been used in various epidemiological and genetic studies.^26–31^ Applications of this high-throughput metabolomics platform has recently been reviewed,^22^ and details of the experimentation have been described elsewhere.^25,32^

For the ALSPAC young participants, metabolic measures were obtained from serum taken at follow-up clinic assessments at the approximate ages of 7, 15, and 17 years. In total, 7176 participants had at least one measurement, with 1453 measured at all 3 ages. For 73 sibling pairs, one child was removed at random before statistical analysis. Samples from the 15- and 17-year follow-up assessments were taken after overnight fast, for those assessed in the morning, and at least 6-hour fast for those assessed after 14.00 hours; samples taken at the 7-year assessment were nonfasted. To allow the maximum sample size for analyses (given the relative stability of fasting versus nonfasting samples^33^), data were taken from all available time points. However, to minimize unnecessary heterogeneity, where participants had repeat measurements, we prioritized those collected under fasted conditions. This led to a final analysis sample with 37.5% nonfasting (sensitivity analyses excluding those nonfasting are provided in the Data Supplement).

Measurements were available for 4530 ALSPAC mothers at a median age of 48 years (with an overlap of 1981 mothers of the young participant sample). All of the samples from the ALSPAC mothers were taken after an overnight fast for those taken in the morning and a minimum 6-hour fast for those taken after 14.00 hours.

Samples were available for 3780 women from the BWHHS at baseline assessment (age 60–79 years) after an overnight fast for those assessed in the morning and a minimum of 6-hour fast for those assessed after 14.00 hours. Given differences in storage of the samples between studies, 225 metabolites were common in all 3 and are used here.

### Genotyping

Genotyping for the rs138326449 splice variant *APOC3* mutation was performed using KASPAR at KBioscience (www.lgcgenomics.com) for all participants from both studies who had a suitable DNA sample. Genotypes for the leading triglyceride-associated *LPL* singe nucleotide polymorphism (SNP) rs12678919, a downstream intergenic variant in linkage disequilibrium with an SNP with previous evidence of transcriptional regulation,^34^ were extracted from the existing genome-wide common variant data available in ALSPAC.^35^ For BWHHS, the SNP was extracted from the available Metabochip array data.^36^ Standard metrics were used to assess the quality of these data (missingness [>3%], non-European ancestry, and SNPs of minor allele frequency of <1%, call rate of <95%, and Hardy–Weinberg equilibrium [*P*<5×10^−7^]).

### Statistical Analysis

The metabolic measures were inspected for deviations from normality and transformed, when needed, using the natural logarithm plus one to be consistent throughout because some metabolic measures included zero values. For the analysis of the association between the metabolic measures and rs138326449(*APOC3*), we used a linear regression model adjusting for age, sex and, to try to adjust for the nonfasting measurements at age 7, as well as any other differences related to handling and storage of the blood samples, an indicator variable for the phase of measurement where relevant. Primary analysis was undertaken in the ALSPAC young participants. Meta-analyzed results, from a fixed-effects model, of ALSPAC mothers and BWHHS were generated in parallel to those from the ALSAPC offspring. This first approach was taken as the comparison of results from these sample sources permits the confirmation of observed results, but the complications of fasting status, age, and sex may complicate inference drawn from of a meta-analysis across all collections. Where overall meta-analysis was undertaken, we first pooled the ALSPAC mothers and young participants using a linear mixed-effects model to adjust for the pedigree correlation.^37^ Subsequently, we used a fixed-effect meta-analysis to combine the results of the pooled ALSPAC sample with the BWHHS women. To address the uncertainty caused by heterogeneity, we also used a random effects meta-analysis model, but because of the small number of study samples available, we consider this as a sensitivity analysis of the main fixed-effects results.^38^ We used the Benjamini and Yekutieli false discovery rate procedure under dependency^39^ to adjust the *P* values of the confirmation analysis for the associations reaching the 0.05 *P* value threshold in the discovery sample and all meta-analysis results for multiple testing as implemented in the p.adjust package in R.

It is assumed that the APOC3 acts on triglyceride and VLDL largely through inhibition of LPL.^12,17^ We tested whether the *APOC3* variant associations with metabolites could be explained by the inhibition of LPL by using the genetic variant *LPL*(rs12678919) as a proxy of LPL protein levels.^18^ We estimated the predicted effect of *APOC3* on the metabolites if this was exclusively through LPL by looking at the ratio of SNP associations between *LPL*(rs12678919) and the focus variant here *APOC3*(rs138326449). The model used the mean of the ratios of the effects of *APOC3* and *LPL* genetic variants on each metabolite to obtain an estimate of the LPL-mediated effects of APOC3. To avoid the inclusion of metabolic measures that do not follow the assumed model of APOC3 action, we made use of the 25% trimmed mean as a true estimate of the effect of APOC3 on LPL. We bootstrapped the sample 1000 times to obtain the standard error of the mean of the ratios. The predicted effect of *APOC3* on each metabolite was estimated as the product of the mean of the ratios and the *LPL* effect per metabolite, whereas the predicted confidence intervals were estimated taking into account the standard errors of both the *LPL* estimates and of the mean of the ratios. The absence of overlap in the coefficients between the predicted and observed *APOC3* estimates were considered as evidence for an effect of APOC3 not mediated by its inhibition of LPL. We followed the same procedure in each of the parallel studies and combined their estimates using a fixed-effects meta-analysis weighted by their sample size.

Analyses were undertaken in Stata (Stata Statistical Software: Release 13. College Station, TX: StataCorp LP) and R 3.1.0^40^ and plots prepared using ggplot2.^41^

## Results

*APOC3*(rs138326449) was present with minor allele frequencies of 0.20% to 0.28% in the 3 studies and adhered to Hardy–Weinberg equilibrium. *LPL*(rs12678919) polymorphism was more common with a minor allele frequencies of 9.2% to 10.6% across the 3 studies. Key characteristic of participants from each study are shown in Table 1.

**Table 1.**
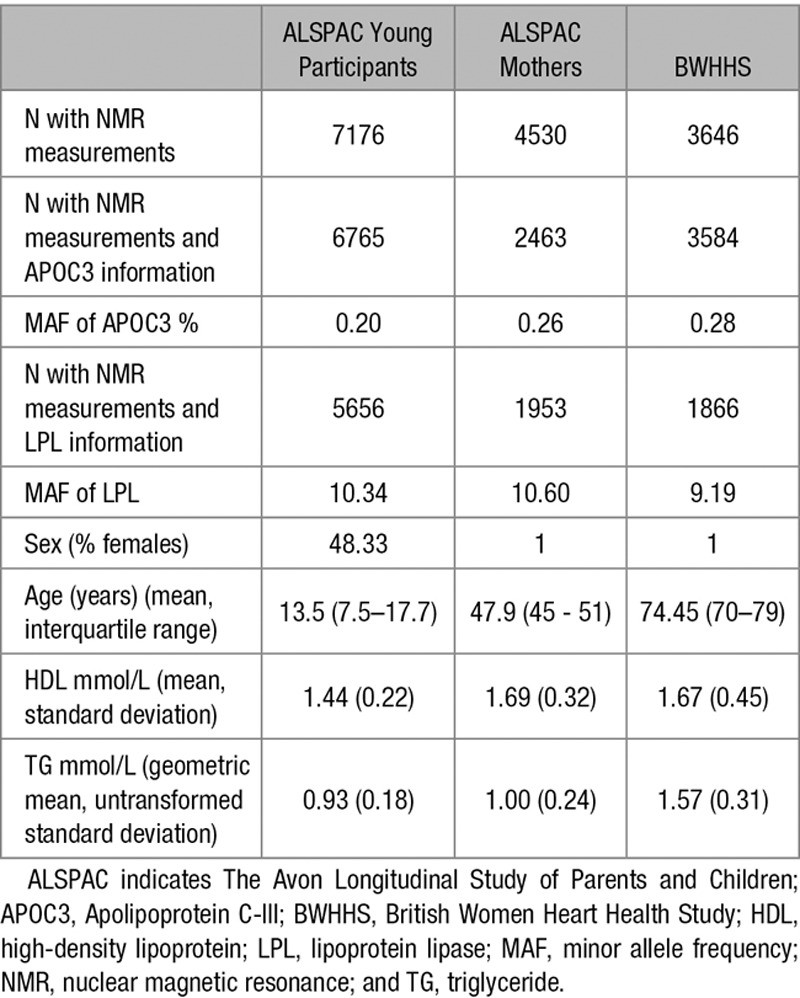
Numbers of Individuals Measured and Key Characteristic of Samples Analyzed

**Figure 1.**
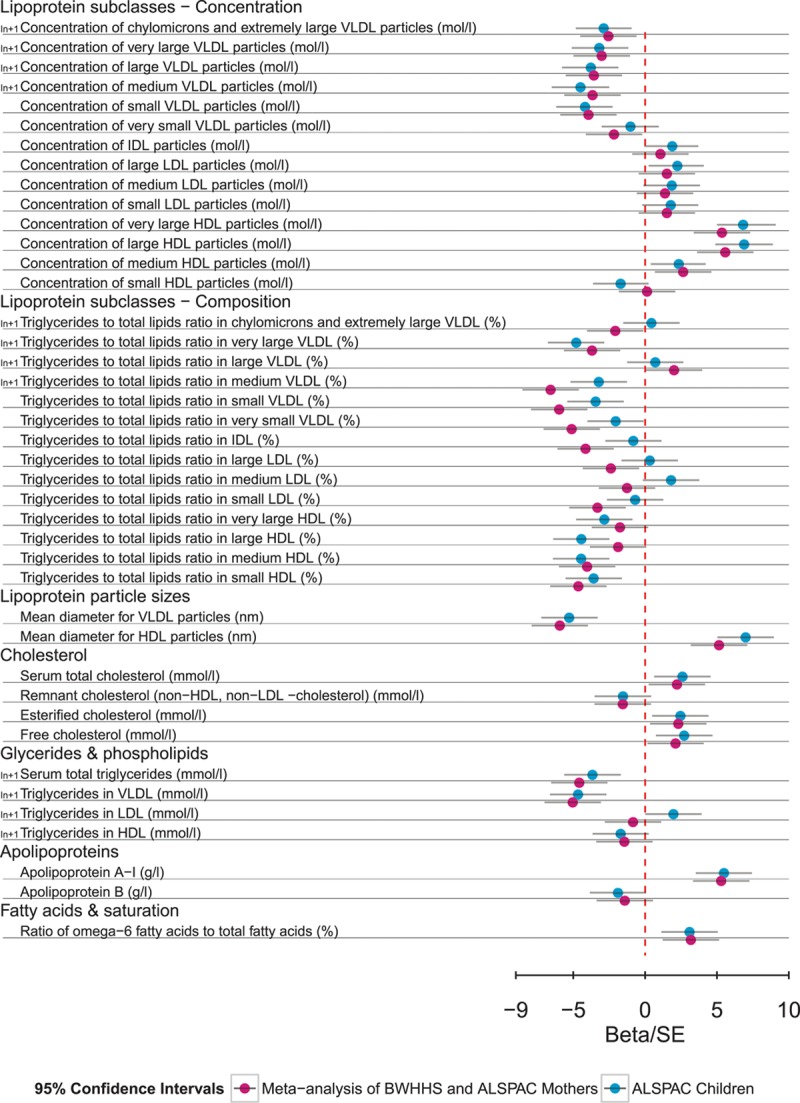
*APOC3*(rs138326449) associations with selected metabolic measures in plasma in ALSPAC young participants and BWHHS–ALSPAC mothers in Beta/SE units. The variant is associated predominately with very low–density lipoprotein (VLDL) and high-density lipoprotein (HDL) concentration and composition, as well as particle size, cholesterol measures, and fatty acids. Multiple associations per particle are represented by a single entry. Information on the transformation used is also provided. Estimates and confidence intervals were scaled by the standard error of each measurement. Plot of the association of all 225 measured metabolites is given in Figure I in the Data Supplement. A detailed list of effect sizes and *P* values for all measures is given Table I in the Data Supplement. ALSPAC indicates The Avon Longitudinal Study of Parents and Children; BWHHS, the British Women Heart Health Study; HDL, high-density lipoprotein; and IDL, intermediate-density lipoproteins.

Of the 225 metabolic measures available in all 3 studies, when analyzed in the ALSPAC young participants, 134 showed nominal evidence of association with *APOC3*(rs138326449; *P*≤0.05). *APOC3*(rs138326449) was associated with a decrease in triglyceride concentration (−0.11 mmol/L of geometric mean, 95% confidence interval −0.16 to −0.05 mmol/L; *P*=2.57×10^–4^) and an increase of HDL (0.26 mmol/L, 95% confidence interval 0.18–0.34 mmol/L; *P*=2.4×10^–6^). More generally, *APOC3*(rs138326449) showed an effect on a broad range of measures reflecting the circulating levels and lipid composition of VLDL and HDL particles (Figure 1). A full table of results for all 225 measures can be found in Table I in the Data Supplement. A comparison of the results from the mixed fasting–nonfasting analysis used and analysis based on fasting and nonfasting measurements showed broadly similar association profiles, though there were differences in the nonfasting samples for specific measures of small HDL, very large VLDL, large LDL, remnant cholesterol, fatty acids, and glutamine (Table II in the Data Supplement).

In meta-analysis results of the ALSPAC mothers and BWHHS samples, 124 metabolic measures showed nominal evidence of association with *APOC3*(rs138326449) (*P*≤0.05; Table I in the Data Supplement), supporting 81 of the signals seen in the ALSPAC younger participants after adjustment for multiple testing (Table III in the Data Supplement). For both triglyceride and HDL, associations were stronger, but consistent with the young participant results (−0.23 mmol/L of geometric mean, 95% confidence interval −0.34 to −0.12 mmol/L; *P*=4.6×10^–6^ and 0.4191 mmol/L, 95% confidence interval 0.28–0.56 mmol/L; *P*=1.74×10^–9^, respectively). In addition to the VLDL and HDL measures of concentration and composition, evidence for an increase of the ratio of ω-6, polyunsaturated and monounsaturated fatty acids to total fatty acid, were also confirmed. When all 3 samples were considered together, 118 associations were observed after adjustment for multiple testing, with 88 also showing evidence of association in the random-effects model. Associations in ALSPAC young participants, meta-analysis of ALSPAC mothers and BWHHS, and the meta-analysis of all 3 samples are presented in Figure I in the Data Supplement, with details for each association in Table I in the Data Supplement.

**Figure 2.**
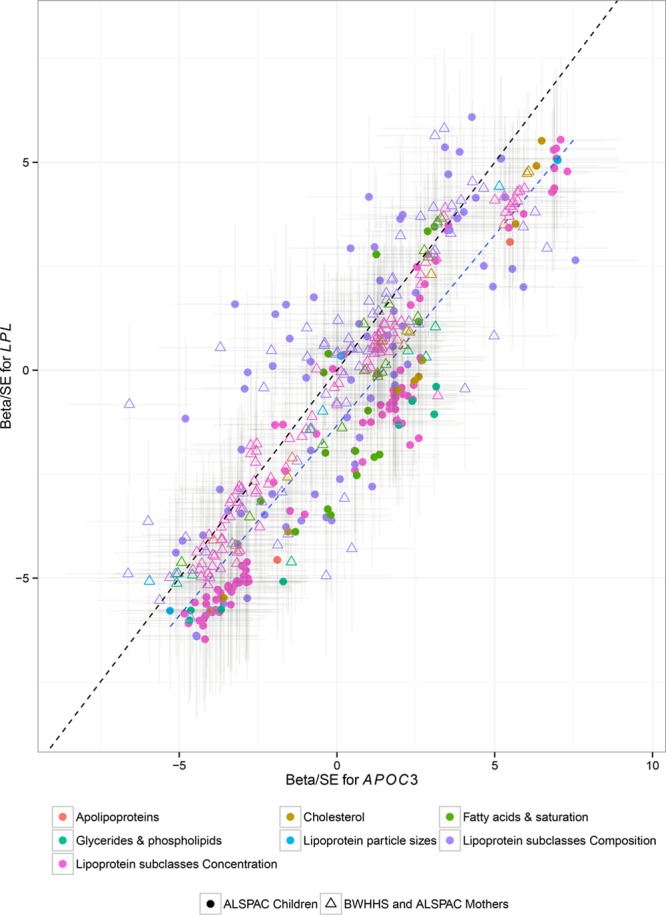
*APOC3*(rs138326449) and *LPL*(rs12678919) correlation of their respective associations with metabolic measures in plasma. The observed effects of *LPL*(rs12678919) are similar to those seen with *APOC3*(rs138326449). The black line is the line of perfect fit, whereas the blue line is the correlation between the 2 metabolic profiles of the 2 singe nucleotide polymorphism (SNPs) with slope equal to 0.87 for the ASPAC young participants. Estimates and confidence intervals were scaled by the standard error of each measurement. A detailed list of the association measures for all metabolites and the *LPL*(rs12678919) is given in Table II in the Data Supplement and plotted in Figure II in the Data Supplement. ALSPAC indicates The Avon Longitudinal Study of Parents and Children; and BWHHS, the British Women Heart Health Study.

The pattern of associations between *LPL* (rs12678919) and the metabolic measures was very similar to that observed for *APOC3* (rs138326449; Figure 2). A Pearson’s correlation test between the coefficients of *LPL* (rs12678919), scaled by their SE, and those obtained for the association of the metabolites with *APOC3* (rs138326449) in the ALSPAC young participants shows strong correlation with *r*=0.88. In ALSPAC young participants, a total of 126 metabolic measures showed nominal evidence of association with *LPL* (rs12678919; *P*≤0.05; Table IV in the Data Supplement). Of these associations, 90 were confirmed in the meta-analysis of ALSPAC mothers and BWHHS samples (false discovery rate adjusted *P*≤0.05; Table V in the Data Supplement). The meta-analysis of all 3 samples revealed 113 associations (false discovery rate adjusted *P*≤0.05), with 75 of them having evidence of association when a mixed-effects model was considered. All association results for the analyses can be seen in Table IV in the Data Supplement and plotted against the discovery effects in Figure II in the Data Supplement.

**Figure 3.**
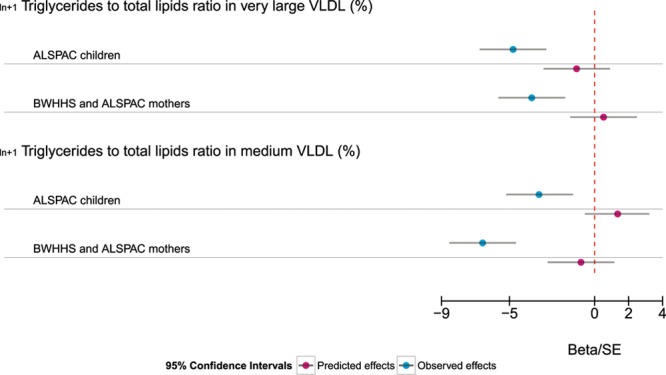
Expected and observed *APOC3*–metabolites associations for the subset of metabolites with a lipoprotein lipase (LPL)–independent effect. The coefficients and confidence interval (CIs) are scaled by the SE of the observed effect. ALSPAC indicates The Avon Longitudinal Study of Parents and Children; BWHHS, the British Women Heart Health Study; and VLDL, very low–density lipoprotein.

There was evidence for *APOC3* effects being mediated through LPL in the majority of the metabolic measures considered. Of the 225 measures tested in the ALSPAC young participants, 6 had no overlapping 95% confidence intervals between the directly observed *APOC3*(rs138326449) effects and those predicted by *LPL*(rs12678919; Table VI in the Data Supplement). The ALSPAC mothers and the BWHHS results confirmed 2 of the suggested 6 APOC3-specific effects as independent from LPL (Table 2 and Figure 3). When all 3 samples were combined, 8 measures showed evidence of nonoverlapping estimates (Table VI in the Data Supplement). Those measures which maintained evidence of an effect outside the action of LPL inhibition were both measures of VLDL composition characterizing the percentage of triglyceride in very large and medium VLDL. All results on the comparison of the LPL predicted and observed APOC3 effects are provided in Table VI in the Data Supplement.

**Table 2.**
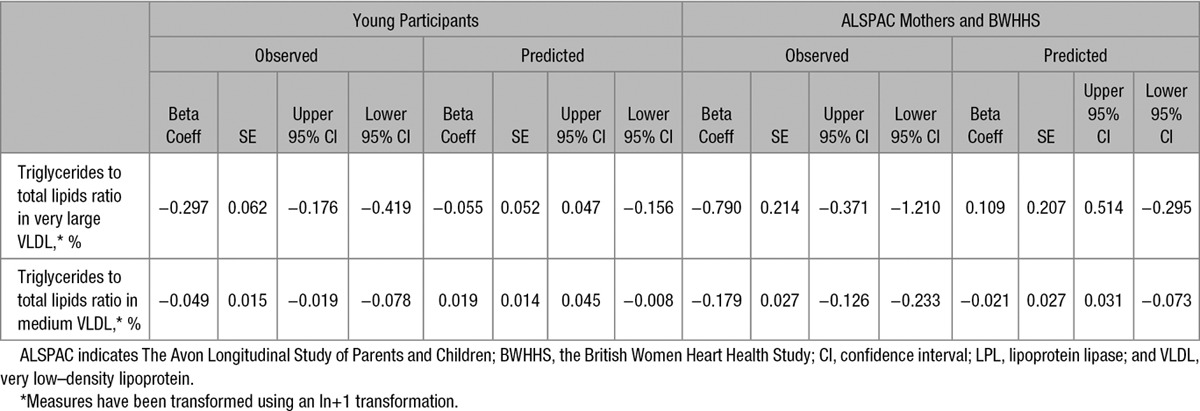
Observed and Predicted Effects of APOC3 on Metabolic Measures With Evidence of an LPL-Independent Mechanism

## Discussion

Using detailed measures of lipoprotein subclass concentration and composition and several other metabolic measures, we provided a profile for the effect of the rare *APOC3* (rs138326449) splice variant on mothers and young participants from one European population cohort and older adult women from an independent European population cohort. We confirmed the previously reported, but crudely assessed, association with triglyceride, VLDL, and HDL^16^ levels and identified additional associations with VLDL and HDL composition, other cholesterol measures, and fatty acids. Using *LPL* (rs12678919) as a proxy for LPL protein levels, we tested the extent to which APOC3 action on lipids is mediated through LPL inhibition.^12,17^ Our results suggest that the great majority of the APOC3 effects are in line with the assumed mode of action through LPL; the composition of very large and medium VLDL particles involve other mechanisms in addition to LPL. Although the pathways involved have been previously studied in model organisms and in vitro experiments,^21^ here we present an epidemiological view of triglyceride metabolism in relation to APOC3 based on extensive metabolite measurements, many of which have not been previously studied in large epidemiological studies. Biological interpretation of the identified associations should thus be in the context of previously accumulated evidence in other experimental systems, many of which we were able to replicate here as also operating in humans.

Although the associations with total VLDL, HDL, and triglyceride levels were in the same direction as those in previous studies,^10,15,16,42^ their magnitude of effect was lower in the young participants. We did not find evidence for the associations of *APOC3* (rs138326449) with either intermediate-density lipoproteins or LDL concentration or composition, with previous studies reporting contradictory effects on LDL for the rare *APOC3* mutations^15^ and its mRNA inhibition.^43^ Consideration of the fasting and nonfasting individuals in the young participants sample separately was consistent with the overall results of no association.

The greater resolution provided by the detail measurement of the lipoprotein subclasses shows that the effects of the splice variant on VLDL and HDL can be seen in almost the entire spectrum of their size. A highly similar pattern can be seen in the *LPL* associations with the metabolic measures. Also, both have an effect on the diameter of VLDL and HDL particles, with the rare allele associated with a decrease in the diameter of VLDL and an increase in the diameter of HDL particles in serum. For VLDL, lipoprotein kinetic studies have shown that the different size VLDL particles are metabolically heterogeneous,^44^ with large subfractions generating remnants that persist in circulation, whereas smaller VLDL particles are rapidly and efficiently converted to LDL,^44^ which agrees with our observations especially for the small VLDL and LDL measures.

Although the assumed mode of effect of APOC3 on triglyceride levels and triglyceride-rich lipoproteins is because of impeded lipolytic conversion and hepatic clearance, in vivo and in vitro evidence point toward an additional role of APOC3 in the production of high triglyceride content VLDL (reviewed in Yao and Wang^21^). Our comparison of the *APOC3* and *LPL* association revealed that the composition of medium and very large VLDL is not fully predicted by the action of APOC3 through LPL. Studies show that *APOC3* expression promotes the assembly and secretion of the bigger triglyceride-rich VLDL from hepatocytes through the mobilization of endoplasmic reticulum/Golgi microsomes triglyceride for VLDL assembly.^45^ This intracellular mechanism is manifest under conditions of insulin resistance or hypertriglyceridemia,^21^ though our results suggest it also operates under normal conditions. Different structural changes in the APOC3 protein, either in the N or C terminals, affect the assembly and secretion of larger VLDL particles in different ways^46,47^ but have no effect on the triglyceride-poor smaller VLDL particles.

Our observations of a APOC3 LPL-independent triglyceride-related pathway agree with Gaudet et al,^48^ testing the effects of an *APOC3* mRNA inhibitor on familial chylomicronemia syndrome sufferers. In this case, deficiency in LPL leads to severe hypertriglyceridemia, which can result in recurrent and potentially fatal pancreatitis. When 3 patients were given an *APOC3* inhibitor that lowered their APOC3 levels, a reduction of triglyceride was observed.^48^ Similar results were obtained in patients with severe or uncontrolled hypertriglyceridemia.^43^ Although we did not find an LPL-independent effect on total serum triglyceride, either because of lack of statistical power or because of the differences between hypertriglyceridemia patients and the samples available here representing the general population, the availability of more refined measures of triglyceride concentration in specific subclasses permitted the identification of the likely mechanism responsible for the effect of APOC3 inhibition. Our results point toward changes in the composition of VLDL and its proportion of triglyceride though an intrahepatic pathway, rather than a mechanism involving changes in triglyceride absorption.

Our study has several limitations, mainly in relation to the differences in age and sex between the 3 study samples and the mix of fasting and nonfasting status in ALSPAC children. For these reasons, the ALSPAC mothers and BWHHS samples were only considered as able to confirm the common observed associations, with false positives indinguishable from heterogeneity between the samples because of age and sex for the discordant result. Fasting status in the ALSPAC young participants was addressed through a sensitivity analysis excluding nonfasting individuals, with no evidence of an effect that can change our conclusions. Finally, the low number of the rare APOC3(rs138326449) variant carriers might have contributed to the no identification of true associations because of low statistical power, especially in the proportional modeling part of our work.

To summarize, we were able to refine and characterize the effects of the newly discovered *APOC3*(rs138326449) loss of function mutation in lipoprotein metabolism and its potential to affect triglyceride levels. We also characterized the effects of the GWAS lead signal in the area of *LPL* rs12678919 and compared its action to that of the APOC3 variant. Our findings suggest that the APOC3 variant has a wide range of actions on lipids and fatty acids beyond its known effect on triglyceride and HDL. Although our novel analyses suggest that much of the action of APOC3 on lipids is mediated via LPL action, as hypothesized, a parallel intracellular mechanism previously only observed in model organisms and cell cultures under conditions mimicking pathophysiological disorders also seem to be relevant for the composition of VLDL particles. Our results support the results of clinical trials on LPL-deficient patients for ISIS 304801, an antisense oligonucleotide inhibitor of *APOC3* mRNA and, thus, illustrate the possible use of such approaches as a relatively quick and low-cost tool in the evaluation of drug targets.

## Acknowledgments

We are extremely grateful to all the families who took part in this study, the midwives for their help in recruiting them, and the whole ALSPAC team, which includes interviewers, computer and laboratory technicians, clerical workers, research scientists, volunteers, managers, receptionists, and nurses. This publication is the work of the authors, and Drs Drenos and Timpson will serve as guarantors for the contents of this article. GWAS data were generated by Sample Logistics and Genotyping Facilities at the Wellcome Trust Sanger Institute and LabCorp (Laboratory Corportation of America) using support from 23andMe.

## Sources of Funding

The UK Medical Research Council and the Wellcome Trust (Grant ref: 102215/2/13/2) and the University of Bristol provide core support for ALSPAC. Grants from the British Heart Foundation (SP/07/008/24066) and Wellcome Trust (WT092830M and WT088806) funded data collection from the ALSPAC mothers. The British Women’s Heart and Health Study has been supported by funding from the British Heart Foundation (BHF; grant PG/13/66/304422). Drs Drenos, Timpson, Davey Smith, and Lawlor all work in a Unit receiving funds from the UK Medical Research Council (MC_UU_12013/1–9). Dr Lawlor is a UK NIH Research Senior Investigator (NF-SI-0611-10196). Dr Würtz is funded by the Finnish Diabetes Research Foundation and the Novo Nordisk Foundation. Dr Kettunen was supported from the Academy of Finland (grant number 283045). The quantitative serum nuclear magnetic resonance (NMR) metabolomics platform and its development have been supported by the Academy of Finland, TEKES (the Finnish Funding Agency for Technology and Innovation), the Sigrid Juselius Foundation, the Novo Nordisk Foundation, the Finnish Diabetes Research Foundation, the Paavo Nurmi Foundation, and the strategic and infrastructural research funding from the University of Oulu, Finland, as well as by the British Heart Foundation, the Wellcome Trust, and the Medical Research Council, UK. The views expressed in this article are those of the authors and not necessarily any funding body. The funders did not have any influence over data collection, analyses, and interpretation of findings or writing of this article.

## Disclosures

A.J. Kangas and Drs Soininen, Würtz, Kettunen, and Ala-Korpela are shareholders of Brainshake Ltd (www.brainshake.fi), a company offering NMR-based metabolite profiling. A. Kangas and Drs Soininen, Würtz, and Kettunen report employment and consulting for Brainshake Ltd. The other authors report no conflicts.

## Supplementary Material

## CLINICAL PERSPECTIVE

Plasma triglycerides are robustly associated with atherosclerosis and coronary heart disease. Apolipoprotein C-III (APOC3), encoded by the *APOC3* gene, is a common component of triglycerides-rich lipoproteins and plays an important role in their hydrolysis by lipoprotein lipase and their subsequent uptake into hepatocytes. Loss-of-function *APOC3* mutations have been associated with lower levels of triglycerides and decreased coronary heart disease risk. Here we studied a rare *APOC3* variant (rs138326449) in participants of 2 European population cohorts measuring a range of metabolic measures, mainly lipids associated, using a high-throughput nuclear magnetic resonance metabolomics platform. Among others, the mutation was associated with decreased very low–density lipoprotein and increased high-density lipoprotein and a decrease of their triglycerides content. No evidence of association could be established with low-density lipoprotein or other remnant cholesterol measures. Comparison between the effects of the *APOC3* mutation and the *LPL* lead signal from GWAS studies revealed that they have similar metabolic effects. Two measures for the proportion of triglycerides in large and medium very low–density lipoprotein do not completely fit the classic model of APOC3 inhibition of lipoprotein lipase. Our results support, on an epidemiological level, an alternative intrahepatic role for the mobilization of intracellular triglycerides for larger very low–density lipoprotein assembly, as observed in animal models and in vitro experiments under conditions mimicking insulin resistance or hypertriglyceridemia, in healthy individuals. This mechanism may be relevant to the action of the ISIS 304801 antisense oligonucleotide inhibitor of *APOC3* mRNA to lower triglycerides in patients with lipoprotein lipase deficiency or hypertriglyceridemia.
