# Supplementary material for: Metabolic Characterization of a Rare Genetic Variation Within APOC3 and Its Lipoprotein Lipase–Independent Effects
Source: Circ Cardiovasc Genet. 2016 Jun 21;9(3):231–9. doi: 10.1161/CIRCGENETICS.115.001302 (PMC4920206; doi:10.1161/CIRCGENETICS.115.001302)
Supplement: Supplementary file 1 [file hcg-9-231-s001.pdf]

SUPPLEMENTAL MATERIAL

Supplemental Figure 1: *APOC3*(rs138326449) associations with all the metabolic measures considered in plasma in the ALSPAC young participants, the ALSPAC mothers, BWHHS, BWHHS – ALSPAC mothers combined estimates and the meta-analysis of all three samples taking into account familial relationships between the ALSPAC mothers and children in Beta/SE units. A detailed list of effect sizes and p-values for all measures can be seen in **Table S1**.

Supplemental Figure 2: *LPL*(rs12678919) associations with all the metabolic measures considered in plasma in the ALSPAC young participants, the ALSPAC mothers, BWHHS, BWHHS – ALSPAC mothers combined estimates and the meta-analysis of all three samples taking into account familial relationships between the ALSPAC mothers and children in Beta/SE units. A detailed list of effect sizes and p-values for all measures can be seen in **Table S4**.

Supplemental Table 1 (See Supplemental Excel File): Full table of results for all 225 measures and their association with the *APOC3* (rs138326449) variant. Sample size (N), Beta coefficient per allele (Beta coef.), Standard error of the estimate (SE), lower and upper 95% confidence intervals (Upper 95% CI , Lower 95% CI respectively), p-value of linear model (P-value), and R^2 (R-square) are given for each of the three samples and their combinations considered. Meta-analysis of all three studies includes a combined ASLAPC sample taking into account the relationship between mothers and children meta-analysed with the BWHHS estimates. P-values for the meta-analysis studies adjusted with the false discovery rate considering all 225 tests.

Supplemental Table 2 (See Supplemental Excel File): Comparison between the results of fasting, non-fasting and combined ALSPAC children samples for all 225 metabolic measures considered and their association with the *APOC3* (rs138326449) variant.

Supplemental Table 3 (See Supplemental Excel File): Beta coefficients and p-values for the metabolic measures with evidence of association of association with the *APOC3* (rs138326449) variant in ALSPAC children and their respective values in the combined ALSPAC mothers-BWHHS meta-analysis with p-values adjusted for the false discovery rate of the 134 tests considered.

Supplemental Table 4 (See Supplemental Excel File): Full table of results for all 225 measures and their association with the *LPL* (rs12678919) polymorphism. Sample size (N), Beta coefficient per allele (Beta coef.), Standard error of the estimate (SE), lower and upper 95% confidence intervals (Upper 95% CI , Lower 95% CI respectively), p-value of linear model (P-value), and R^2 (R-square) are given for each of the three samples and their combinations considered. Meta-analysis of all three studies includes a combined ASLAPC sample taking into account the relationship between mothers and children meta-analysed with the BWHHS estimates. P-values for the meta-analysis studies adjusted with the false discovery rate considering all 225 tests.

Supplemental Table 5 (See Supplemental Excel File): Beta coefficients and p-values for the metabolic measures with evidence of association of association with the *LPL* (rs12678919) polymorphism in ALSPAC children and their respective values in the combined ALSPAC mothers-BWHHS meta-analysis with p-values adjusted for the false discovery rate of the 126 tests considered.

Supplemental Table 6 (See Supplemental Excel File): Full table of expected and observed effects of all 225 measures under the model of APOC3 inhibition of TRLs hydrolysis by LPL for ALSPAC children and the meta-analysed ALSPAC mothers-BWHHS estimates. The differences between observed and expected were meta-analysed and the overall difference and p-value from a fixed effects meta-analysis are also presented.

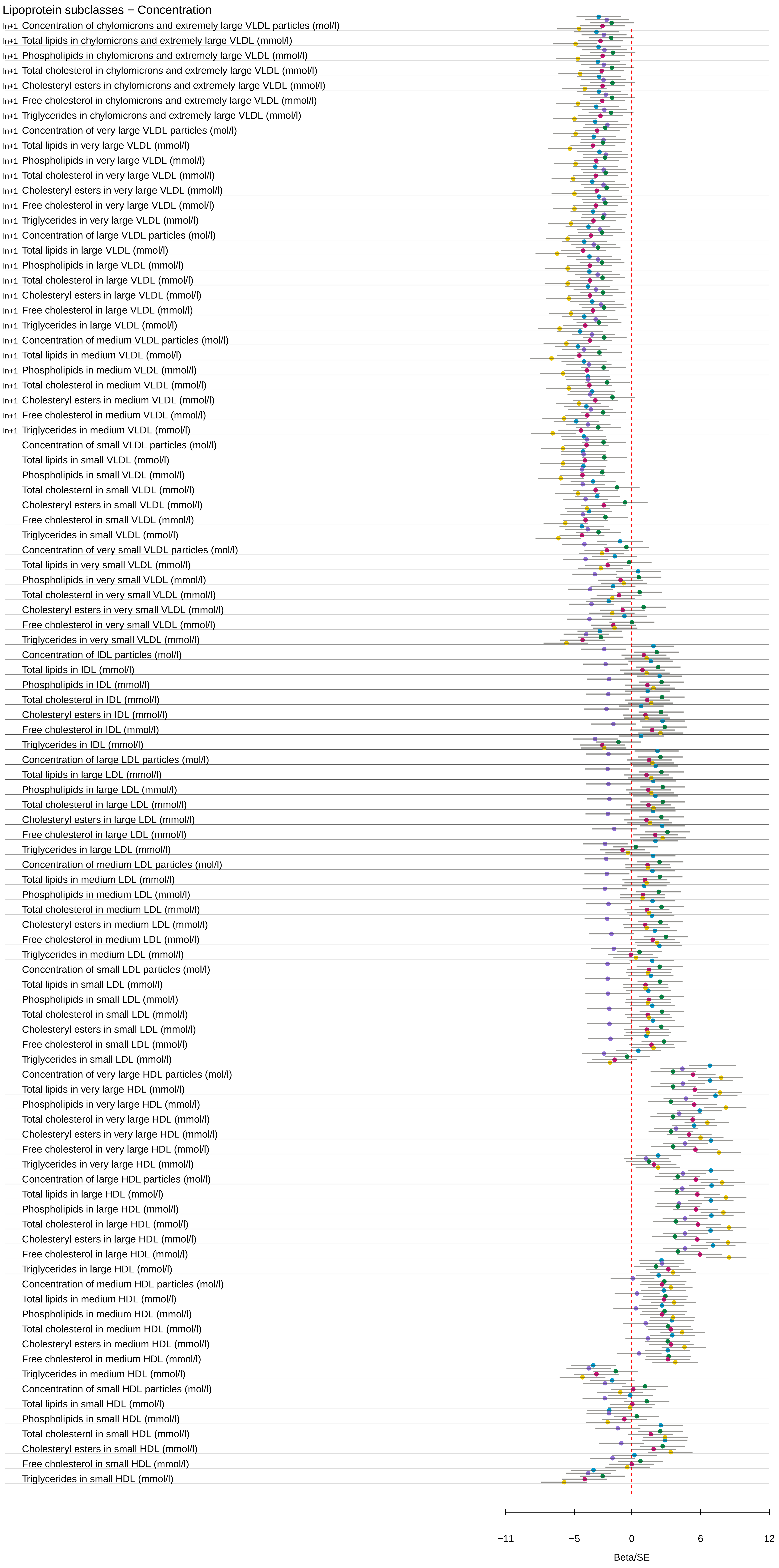
Lipoprotein subclasses − Concentration
ln+1 Concentration of chylomicrons and extremely large VLDL particles (mol/l)
ln+1 Total lipids in chylomicrons and extremely large VLDL (mmol/l)
ln+1 Phospholipids in chylomicrons and extremely large VLDL (mmol/l)
ln+1 Total cholesterol in chylomicrons and extremely large VLDL (mmol/l)
ln+1 Cholesteryl esters in chylomicrons and extremely large VLDL (mmol/l)
ln+1 Free cholesterol in chylomicrons and extremely large VLDL (mmol/l)
ln+1 Triglycerides in chylomicrons and extremely large VLDL (mmol/l)
ln+1 Concentration of very large VLDL particles (mol/l)
ln+1 Total lipids in very large VLDL (mmol/l)
ln+1 Phospholipids in very large VLDL (mmol/l)
ln+1 Total cholesterol in very large VLDL (mmol/l)
ln+1 Cholesteryl esters in very large VLDL (mmol/l)
ln+1 Free cholesterol in very large VLDL (mmol/l)
ln+1 Triglycerides in very large VLDL (mmol/l)
ln+1 Concentration of large VLDL particles (mol/l)
ln+1 Total lipids in large VLDL (mmol/l)
ln+1 Phospholipids in large VLDL (mmol/l)
ln+1 Total cholesterol in large VLDL (mmol/l)
ln+1 Cholesteryl esters in large VLDL (mmol/l)
ln+1 Free cholesterol in large VLDL (mmol/l)
ln+1 Triglycerides in large VLDL (mmol/l)
ln+1 Concentration of medium VLDL particles (mol/l)
ln+1 Total lipids in medium VLDL (mmol/l)
ln+1 Phospholipids in medium VLDL (mmol/l)
ln+1 Total cholesterol in medium VLDL (mmol/l)
ln+1 Cholesteryl esters in medium VLDL (mmol/l)
ln+1 Free cholesterol in medium VLDL (mmol/l)
ln+1 Triglycerides in medium VLDL (mmol/l)
Concentration of small VLDL particles (mol/l)
Total lipids in small VLDL (mmol/l)
Phospholipids in small VLDL (mmol/l)
Total cholesterol in small VLDL (mmol/l)
Cholesteryl esters in small VLDL (mmol/l)
Free cholesterol in small VLDL (mmol/l)
Triglycerides in small VLDL (mmol/l)
Concentration of very small VLDL particles (mol/l)
Total lipids in very small VLDL (mmol/l)
Phospholipids in very small VLDL (mmol/l)
Total cholesterol in very small VLDL (mmol/l)
Cholesteryl esters in very small VLDL (mmol/l)
Free cholesterol in very small VLDL (mmol/l)
Triglycerides in very small VLDL (mmol/l)
Concentration of IDL particles (mol/l)
Total lipids in IDL (mmol/l)
Phospholipids in IDL (mmol/l)
Total cholesterol in IDL (mmol/l)
Cholesteryl esters in IDL (mmol/l)
Free cholesterol in IDL (mmol/l)
Triglycerides in IDL (mmol/l)
Concentration of large LDL particles (mol/l)
Total lipids in large LDL (mmol/l)
Phospholipids in large LDL (mmol/l)
Total cholesterol in large LDL (mmol/l)
Cholesteryl esters in large LDL (mmol/l)
Free cholesterol in large LDL (mmol/l)
Triglycerides in large LDL (mmol/l)
Concentration of medium LDL particles (mol/l)
Total lipids in medium LDL (mmol/l)
Phospholipids in medium LDL (mmol/l)
Total cholesterol in medium LDL (mmol/l)
Cholesteryl esters in medium LDL (mmol/l)
Free cholesterol in medium LDL (mmol/l)
Triglycerides in medium LDL (mmol/l)
Concentration of small LDL particles (mol/l)
Total lipids in small LDL (mmol/l)
Phospholipids in small LDL (mmol/l)
Total cholesterol in small LDL (mmol/l)
Cholesteryl esters in small LDL (mmol/l)
Free cholesterol in small LDL (mmol/l)
Triglycerides in small LDL (mmol/l)
Concentration of very large HDL particles (mol/l)
Total lipids in very large HDL (mmol/l)
Phospholipids in very large HDL (mmol/l)
Total cholesterol in very large HDL (mmol/l)
Cholesteryl esters in very large HDL (mmol/l)
Free cholesterol in very large HDL (mmol/l)
Triglycerides in very large HDL (mmol/l)
Concentration of large HDL particles (mol/l)
Total lipids in large HDL (mmol/l)
Phospholipids in large HDL (mmol/l)
Total cholesterol in large HDL (mmol/l)
Cholesteryl esters in large HDL (mmol/l)
Free cholesterol in large HDL (mmol/l)
Triglycerides in large HDL (mmol/l)
Concentration of medium HDL particles (mol/l)
Total lipids in medium HDL (mmol/l)
Phospholipids in medium HDL (mmol/l)
Total cholesterol in medium HDL (mmol/l)
Cholesteryl esters in medium HDL (mmol/l)
Free cholesterol in medium HDL (mmol/l)
Triglycerides in medium HDL (mmol/l)
Concentration of small HDL particles (mol/l)
Total lipids in small HDL (mmol/l)
Phospholipids in small HDL (mmol/l)
Total cholesterol in small HDL (mmol/l)
Cholesteryl esters in small HDL (mmol/l)
Free cholesterol in small HDL (mmol/l)
Triglycerides in small HDL (mmol/l)
−11
−5
0
6
12
Beta/SE

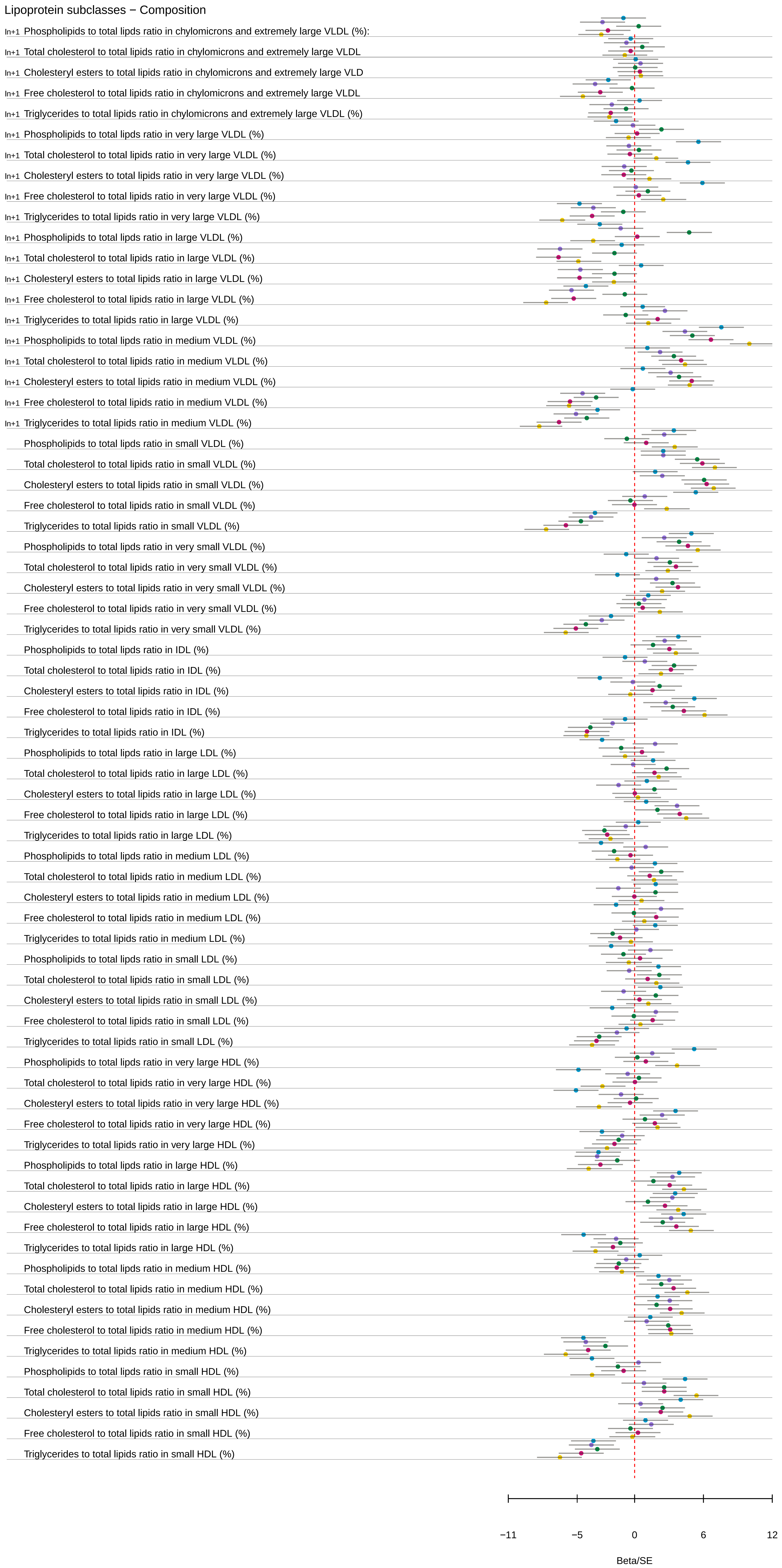
Lipoprotein subclasses − Composition
ln+1 Phospholipids to total lipds ratio in chylomicrons and extremely large VLDL (%):
ln+1 Total cholesterol to total lipids ratio in chylomicrons and extremely large VLDL
ln+1 Cholesteryl esters to total lipids ratio in chylomicrons and extremely large VLD
ln+1 Free cholesterol to total lipids ratio in chylomicrons and extremely large VLDL
ln+1 Triglycerides to total lipids ratio in chylomicrons and extremely large VLDL (%)
ln+1 Phospholipids to total lipds ratio in very large VLDL (%)
ln+1 Total cholesterol to total lipids ratio in very large VLDL (%)
ln+1 Cholesteryl esters to total lipids ratio in very large VLDL (%)
ln+1 Free cholesterol to total lipids ratio in very large VLDL (%)
ln+1 Triglycerides to total lipids ratio in very large VLDL (%)
ln+1 Phospholipids to total lipds ratio in large VLDL (%)
ln+1 Total cholesterol to total lipids ratio in large VLDL (%)
ln+1 Cholesteryl esters to total lipids ratio in large VLDL (%)
ln+1 Free cholesterol to total lipids ratio in large VLDL (%)
ln+1 Triglycerides to total lipids ratio in large VLDL (%)
ln+1 Phospholipids to total lipds ratio in medium VLDL (%)
ln+1 Total cholesterol to total lipids ratio in medium VLDL (%)
ln+1 Cholesteryl esters to total lipids ratio in medium VLDL (%)
ln+1 Free cholesterol to total lipids ratio in medium VLDL (%)
ln+1 Triglycerides to total lipids ratio in medium VLDL (%)
Phospholipids to total lipds ratio in small VLDL (%)
Total cholesterol to total lipids ratio in small VLDL (%)
Cholesteryl esters to total lipids ratio in small VLDL (%)
Free cholesterol to total lipids ratio in small VLDL (%)
Triglycerides to total lipids ratio in small VLDL (%)
Phospholipids to total lipds ratio in very small VLDL (%)
Total cholesterol to total lipids ratio in very small VLDL (%)
Cholesteryl esters to total lipids ratio in very small VLDL (%)
Free cholesterol to total lipids ratio in very small VLDL (%)
Triglycerides to total lipids ratio in very small VLDL (%)
Phospholipids to total lipds ratio in IDL (%)
Total cholesterol to total lipids ratio in IDL (%)
Cholesteryl esters to total lipids ratio in IDL (%)
Free cholesterol to total lipids ratio in IDL (%)
Triglycerides to total lipids ratio in IDL (%)
Phospholipids to total lipds ratio in large LDL (%)
Total cholesterol to total lipids ratio in large LDL (%)
Cholesteryl esters to total lipids ratio in large LDL (%)
Free cholesterol to total lipids ratio in large LDL (%)
Triglycerides to total lipids ratio in large LDL (%)
Phospholipids to total lipds ratio in medium LDL (%)
Total cholesterol to total lipids ratio in medium LDL (%)
Cholesteryl esters to total lipids ratio in medium LDL (%)
Free cholesterol to total lipids ratio in medium LDL (%)
Triglycerides to total lipids ratio in medium LDL (%)
Phospholipids to total lipds ratio in small LDL (%)
Total cholesterol to total lipids ratio in small LDL (%)
Cholesteryl esters to total lipids ratio in small LDL (%)
Free cholesterol to total lipids ratio in small LDL (%)
Triglycerides to total lipids ratio in small LDL (%)
Phospholipids to total lipds ratio in very large HDL (%)
Total cholesterol to total lipids ratio in very large HDL (%)
Cholesteryl esters to total lipids ratio in very large HDL (%)
Free cholesterol to total lipids ratio in very large HDL (%)
Triglycerides to total lipids ratio in very large HDL (%)
Phospholipids to total lipds ratio in large HDL (%)
Total cholesterol to total lipids ratio in large HDL (%)
Cholesteryl esters to total lipids ratio in large HDL (%)
Free cholesterol to total lipids ratio in large HDL (%)
Triglycerides to total lipids ratio in large HDL (%)
Phospholipids to total lipds ratio in medium HDL (%)
Total cholesterol to total lipids ratio in medium HDL (%)
Cholesteryl esters to total lipids ratio in medium HDL (%)
Free cholesterol to total lipids ratio in medium HDL (%)
Triglycerides to total lipids ratio in medium HDL (%)
Phospholipids to total lipds ratio in small HDL (%)
Total cholesterol to total lipids ratio in small HDL (%)
Cholesteryl esters to total lipids ratio in small HDL (%)
Free cholesterol to total lipids ratio in small HDL (%)
Triglycerides to total lipids ratio in small HDL (%)
−11
−5
0
6
12
Beta/SE

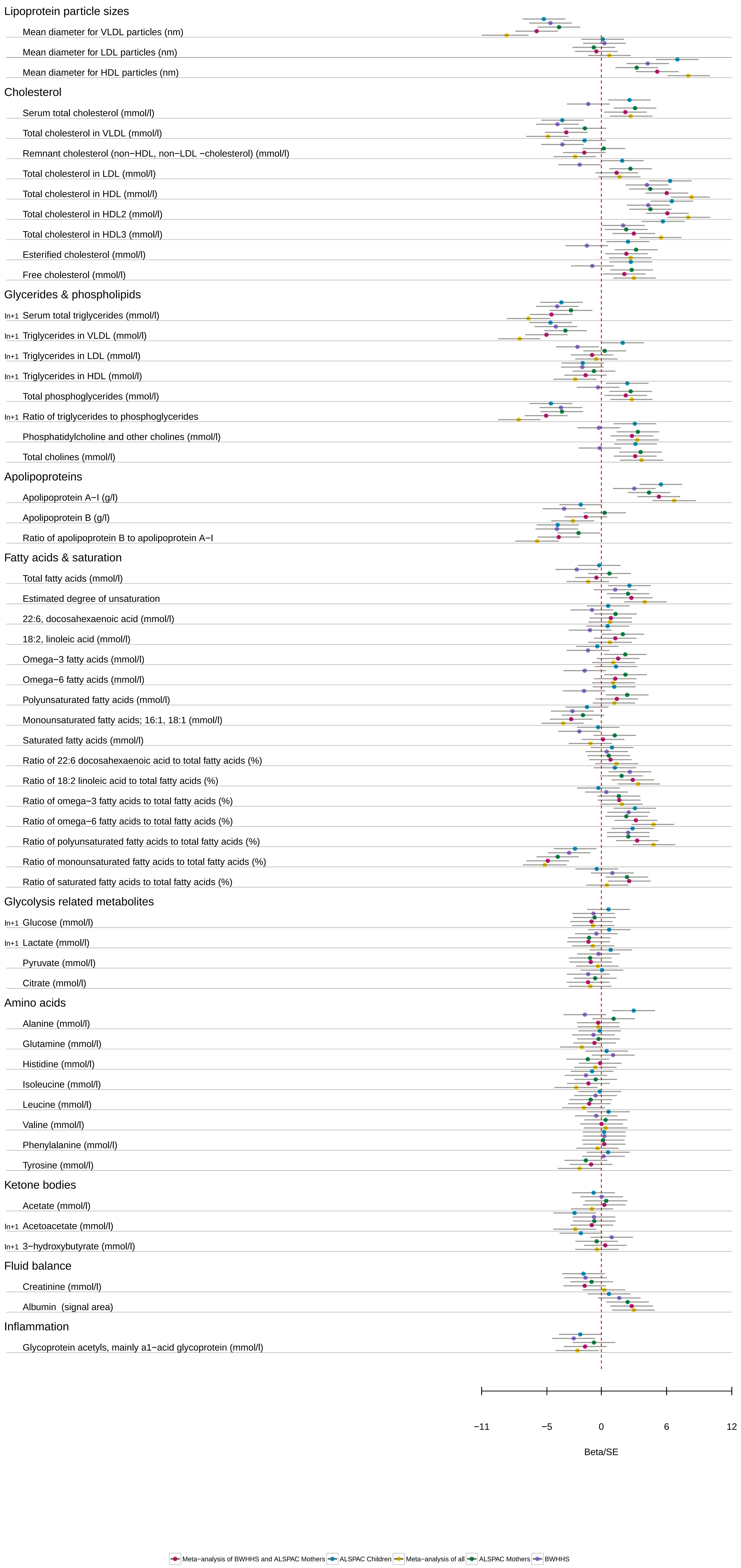
Lipoprotein particle sizes
Mean diameter for VLDL particles (nm)
Mean diameter for LDL particles (nm)
Mean diameter for HDL particles (nm)
Cholesterol
Serum total cholesterol (mmol/l)
Total cholesterol in VLDL (mmol/l)
Remnant cholesterol (non−HDL, non−LDL −cholesterol) (mmol/l)
Total cholesterol in LDL (mmol/l)
Total cholesterol in HDL (mmol/l)
Total cholesterol in HDL2 (mmol/l)
Total cholesterol in HDL3 (mmol/l)
Esterified cholesterol (mmol/l)
Free cholesterol (mmol/l)
Glycerides & phospholipids
ln+1 Serum total triglycerides (mmol/l)
ln+1 Triglycerides in VLDL (mmol/l)
ln+1 Triglycerides in LDL (mmol/l)
ln+1 Triglycerides in HDL (mmol/l)
Total phosphoglycerides (mmol/l)
ln+1 Ratio of triglycerides to phosphoglycerides
Phosphatidylcholine and other cholines (mmol/l)
Total cholines (mmol/l)
Apolipoproteins
Apolipoprotein A−I (g/l)
Apolipoprotein B (g/l)
Ratio of apolipoprotein B to apolipoprotein A−I
Fatty acids & saturation
Total fatty acids (mmol/l)
Estimated degree of unsaturation
22:6, docosahexaenoic acid (mmol/l)
18:2, linoleic acid (mmol/l)
Omega−3 fatty acids (mmol/l)
Omega−6 fatty acids (mmol/l)
Polyunsaturated fatty acids (mmol/l)
Monounsaturated fatty acids; 16:1, 18:1 (mmol/l)
Saturated fatty acids (mmol/l)
Ratio of 22:6 docosahexaenoic acid to total fatty acids (%)
Ratio of 18:2 linoleic acid to total fatty acids (%)
Ratio of omega−3 fatty acids to total fatty acids (%)
Ratio of omega−6 fatty acids to total fatty acids (%)
Ratio of polyunsaturated fatty acids to total fatty acids (%)
Ratio of monounsaturated fatty acids to total fatty acids (%)
Ratio of saturated fatty acids to total fatty acids (%)
Glycolysis related metabolites
ln+1 Glucose (mmol/l)
ln+1 Lactate (mmol/l)
Pyruvate (mmol/l)
Citrate (mmol/l)
Amino acids
Alanine (mmol/l)
Glutamine (mmol/l)
Histidine (mmol/l)
Isoleucine (mmol/l)
Leucine (mmol/l)
Valine (mmol/l)
Phenylalanine (mmol/l)
Tyrosine (mmol/l)
Ketone bodies
Acetate (mmol/l)
ln+1 Acetoacetate (mmol/l)
ln+1 3−hydroxybutyrate (mmol/l)
Fluid balance
Creatinine (mmol/l)
Albumin (signal area)
Inflammation
Glycoprotein acetyls, mainly a1−acid glycoprotein (mmol/l)
−11
−5
0
6
12
Beta/SE
Meta−analysis of BWHHS and ALSPAC Mothers
ALSPAC Children
Meta−analysis of all
ALSPAC Mothers
BWHHS

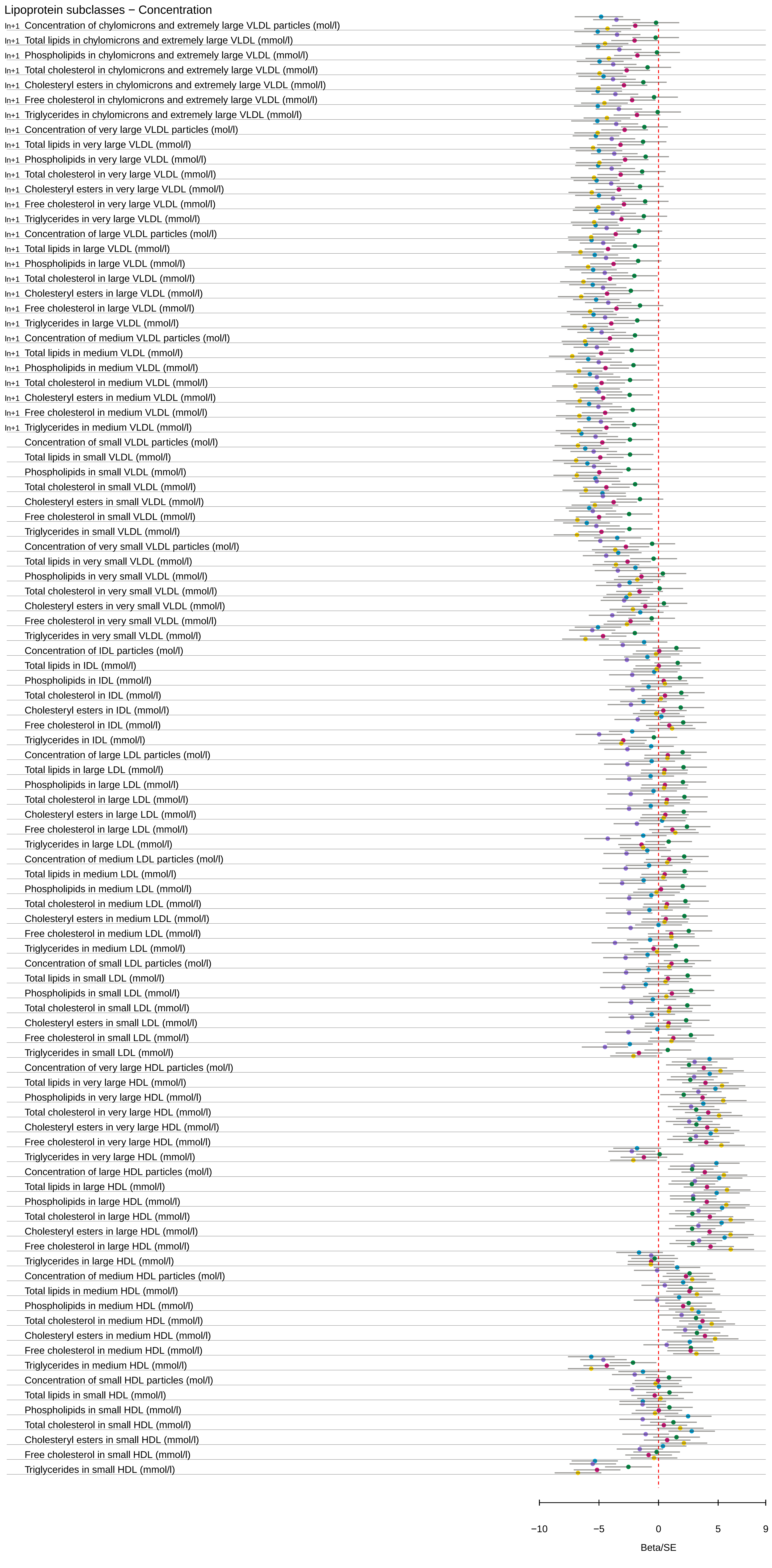
Lipoprotein subclasses − Concentration
ln+1 Concentration of chylomicrons and extremely large VLDL particles (mol/l)
ln+1 Total lipids in chylomicrons and extremely large VLDL (mmol/l)
ln+1 Phospholipids in chylomicrons and extremely large VLDL (mmol/l)
ln+1 Total cholesterol in chylomicrons and extremely large VLDL (mmol/l)
ln+1 Cholesteryl esters in chylomicrons and extremely large VLDL (mmol/l)
ln+1 Free cholesterol in chylomicrons and extremely large VLDL (mmol/l)
ln+1 Triglycerides in chylomicrons and extremely large VLDL (mmol/l)
ln+1 Concentration of very large VLDL particles (mol/l)
ln+1 Total lipids in very large VLDL (mmol/l)
ln+1 Phospholipids in very large VLDL (mmol/l)
ln+1 Total cholesterol in very large VLDL (mmol/l)
ln+1 Cholesteryl esters in very large VLDL (mmol/l)
ln+1 Free cholesterol in very large VLDL (mmol/l)
ln+1 Triglycerides in very large VLDL (mmol/l)
ln+1 Concentration of large VLDL particles (mol/l)
ln+1 Total lipids in large VLDL (mmol/l)
ln+1 Phospholipids in large VLDL (mmol/l)
ln+1 Total cholesterol in large VLDL (mmol/l)
ln+1 Cholesteryl esters in large VLDL (mmol/l)
ln+1 Free cholesterol in large VLDL (mmol/l)
ln+1 Triglycerides in large VLDL (mmol/l)
ln+1 Concentration of medium VLDL particles (mol/l)
ln+1 Total lipids in medium VLDL (mmol/l)
ln+1 Phospholipids in medium VLDL (mmol/l)
ln+1 Total cholesterol in medium VLDL (mmol/l)
ln+1 Cholesteryl esters in medium VLDL (mmol/l)
ln+1 Free cholesterol in medium VLDL (mmol/l)
ln+1 Triglycerides in medium VLDL (mmol/l)
Concentration of small VLDL particles (mol/l)
Total lipids in small VLDL (mmol/l)
Phospholipids in small VLDL (mmol/l)
Total cholesterol in small VLDL (mmol/l)
Cholesteryl esters in small VLDL (mmol/l)
Free cholesterol in small VLDL (mmol/l)
Triglycerides in small VLDL (mmol/l)
Concentration of very small VLDL particles (mol/l)
Total lipids in very small VLDL (mmol/l)
Phospholipids in very small VLDL (mmol/l)
Total cholesterol in very small VLDL (mmol/l)
Cholesteryl esters in very small VLDL (mmol/l)
Free cholesterol in very small VLDL (mmol/l)
Triglycerides in very small VLDL (mmol/l)
Concentration of IDL particles (mol/l)
Total lipids in IDL (mmol/l)
Phospholipids in IDL (mmol/l)
Total cholesterol in IDL (mmol/l)
Cholesteryl esters in IDL (mmol/l)
Free cholesterol in IDL (mmol/l)
Triglycerides in IDL (mmol/l)
Concentration of large LDL particles (mol/l)
Total lipids in large LDL (mmol/l)
Phospholipids in large LDL (mmol/l)
Total cholesterol in large LDL (mmol/l)
Cholesteryl esters in large LDL (mmol/l)
Free cholesterol in large LDL (mmol/l)
Triglycerides in large LDL (mmol/l)
Concentration of medium LDL particles (mol/l)
Total lipids in medium LDL (mmol/l)
Phospholipids in medium LDL (mmol/l)
Total cholesterol in medium LDL (mmol/l)
Cholesteryl esters in medium LDL (mmol/l)
Free cholesterol in medium LDL (mmol/l)
Triglycerides in medium LDL (mmol/l)
Concentration of small LDL particles (mol/l)
Total lipids in small LDL (mmol/l)
Phospholipids in small LDL (mmol/l)
Total cholesterol in small LDL (mmol/l)
Cholesteryl esters in small LDL (mmol/l)
Free cholesterol in small LDL (mmol/l)
Triglycerides in small LDL (mmol/l)
Concentration of very large HDL particles (mol/l)
Total lipids in very large HDL (mmol/l)
Phospholipids in very large HDL (mmol/l)
Total cholesterol in very large HDL (mmol/l)
Cholesteryl esters in very large HDL (mmol/l)
Free cholesterol in very large HDL (mmol/l)
Triglycerides in very large HDL (mmol/l)
Concentration of large HDL particles (mol/l)
Total lipids in large HDL (mmol/l)
Phospholipids in large HDL (mmol/l)
Total cholesterol in large HDL (mmol/l)
Cholesteryl esters in large HDL (mmol/l)
Free cholesterol in large HDL (mmol/l)
Triglycerides in large HDL (mmol/l)
Concentration of medium HDL particles (mol/l)
Total lipids in medium HDL (mmol/l)
Phospholipids in medium HDL (mmol/l)
Total cholesterol in medium HDL (mmol/l)
Cholesteryl esters in medium HDL (mmol/l)
Free cholesterol in medium HDL (mmol/l)
Triglycerides in medium HDL (mmol/l)
Concentration of small HDL particles (mol/l)
Total lipids in small HDL (mmol/l)
Phospholipids in small HDL (mmol/l)
Total cholesterol in small HDL (mmol/l)
Cholesteryl esters in small HDL (mmol/l)
Free cholesterol in small HDL (mmol/l)
Triglycerides in small HDL (mmol/l)
−10
−5
0
5
9
Beta/SE

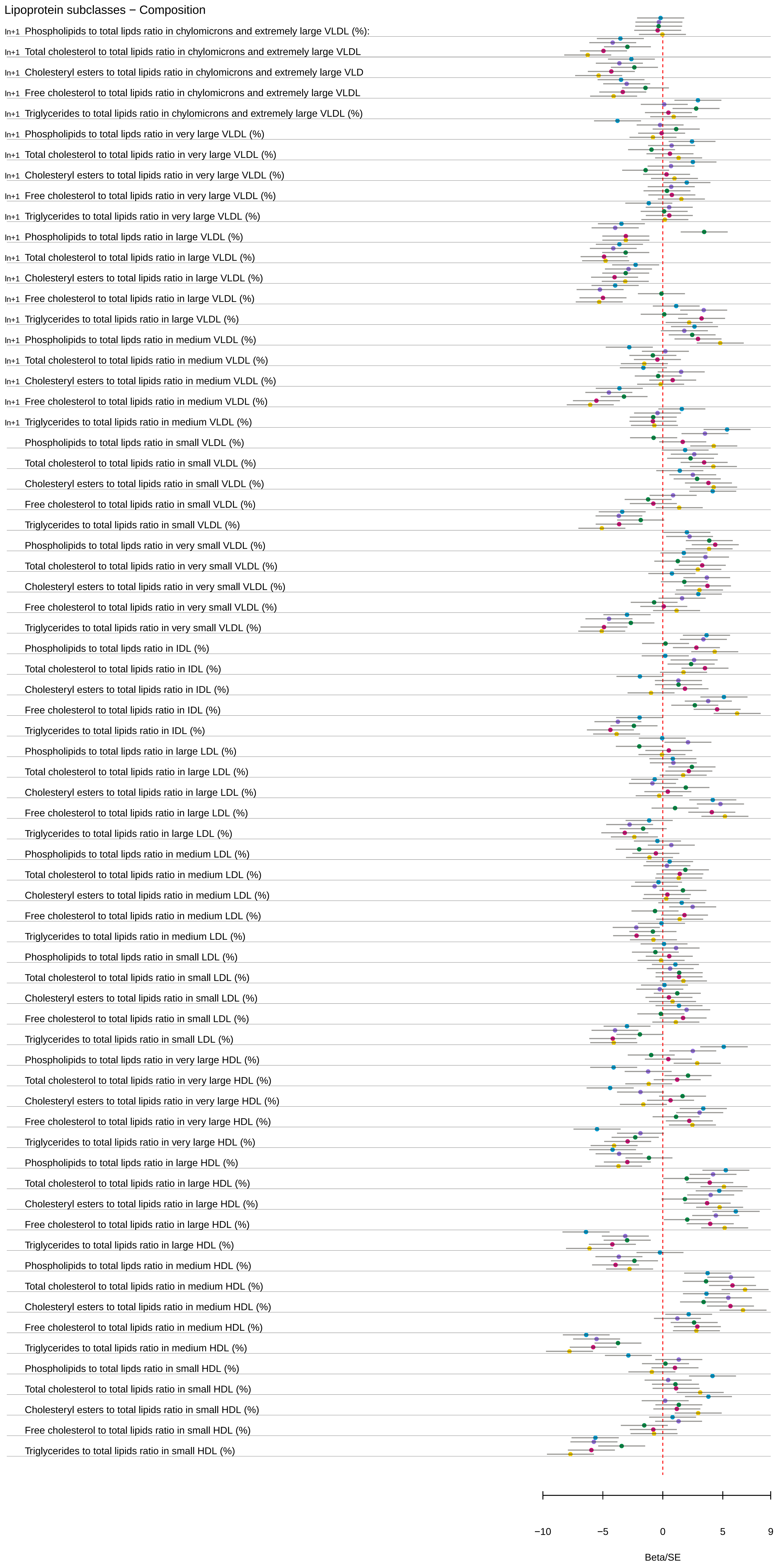
Lipoprotein subclasses − Composition
ln+1 Phospholipids to total lipds ratio in chylomicrons and extremely large VLDL (%):
ln+1 Total cholesterol to total lipids ratio in chylomicrons and extremely large VLDL
ln+1 Cholesteryl esters to total lipids ratio in chylomicrons and extremely large VLD
ln+1 Free cholesterol to total lipids ratio in chylomicrons and extremely large VLDL
ln+1 Triglycerides to total lipids ratio in chylomicrons and extremely large VLDL (%)
ln+1 Phospholipids to total lipds ratio in very large VLDL (%)
ln+1 Total cholesterol to total lipids ratio in very large VLDL (%)
ln+1 Cholesteryl esters to total lipids ratio in very large VLDL (%)
ln+1 Free cholesterol to total lipids ratio in very large VLDL (%)
ln+1 Triglycerides to total lipids ratio in very large VLDL (%)
ln+1 Phospholipids to total lipds ratio in large VLDL (%)
ln+1 Total cholesterol to total lipids ratio in large VLDL (%)
ln+1 Cholesteryl esters to total lipids ratio in large VLDL (%)
ln+1 Free cholesterol to total lipids ratio in large VLDL (%)
ln+1 Triglycerides to total lipids ratio in large VLDL (%)
ln+1 Phospholipids to total lipds ratio in medium VLDL (%)
ln+1 Total cholesterol to total lipids ratio in medium VLDL (%)
ln+1 Cholesteryl esters to total lipids ratio in medium VLDL (%)
ln+1 Free cholesterol to total lipids ratio in medium VLDL (%)
ln+1 Triglycerides to total lipids ratio in medium VLDL (%)
Phospholipids to total lipds ratio in small VLDL (%)
Total cholesterol to total lipids ratio in small VLDL (%)
Cholesteryl esters to total lipids ratio in small VLDL (%)
Free cholesterol to total lipids ratio in small VLDL (%)
Triglycerides to total lipids ratio in small VLDL (%)
Phospholipids to total lipds ratio in very small VLDL (%)
Total cholesterol to total lipids ratio in very small VLDL (%)
Cholesteryl esters to total lipids ratio in very small VLDL (%)
Free cholesterol to total lipids ratio in very small VLDL (%)
Triglycerides to total lipids ratio in very small VLDL (%)
Phospholipids to total lipds ratio in IDL (%)
Total cholesterol to total lipids ratio in IDL (%)
Cholesteryl esters to total lipids ratio in IDL (%)
Free cholesterol to total lipids ratio in IDL (%)
Triglycerides to total lipids ratio in IDL (%)
Phospholipids to total lipds ratio in large LDL (%)
Total cholesterol to total lipids ratio in large LDL (%)
Cholesteryl esters to total lipids ratio in large LDL (%)
Free cholesterol to total lipids ratio in large LDL (%)
Triglycerides to total lipids ratio in large LDL (%)
Phospholipids to total lipds ratio in medium LDL (%)
Total cholesterol to total lipids ratio in medium LDL (%)
Cholesteryl esters to total lipids ratio in medium LDL (%)
Free cholesterol to total lipids ratio in medium LDL (%)
Triglycerides to total lipids ratio in medium LDL (%)
Phospholipids to total lipds ratio in small LDL (%)
Total cholesterol to total lipids ratio in small LDL (%)
Cholesteryl esters to total lipids ratio in small LDL (%)
Free cholesterol to total lipids ratio in small LDL (%)
Triglycerides to total lipids ratio in small LDL (%)
Phospholipids to total lipds ratio in very large HDL (%)
Total cholesterol to total lipids ratio in very large HDL (%)
Cholesteryl esters to total lipids ratio in very large HDL (%)
Free cholesterol to total lipids ratio in very large HDL (%)
Triglycerides to total lipids ratio in very large HDL (%)
Phospholipids to total lipds ratio in large HDL (%)
Total cholesterol to total lipids ratio in large HDL (%)
Cholesteryl esters to total lipids ratio in large HDL (%)
Free cholesterol to total lipids ratio in large HDL (%)
Triglycerides to total lipids ratio in large HDL (%)
Phospholipids to total lipds ratio in medium HDL (%)
Total cholesterol to total lipids ratio in medium HDL (%)
Cholesteryl esters to total lipids ratio in medium HDL (%)
Free cholesterol to total lipids ratio in medium HDL (%)
Triglycerides to total lipids ratio in medium HDL (%)
Phospholipids to total lipds ratio in small HDL (%)
Total cholesterol to total lipids ratio in small HDL (%)
Cholesteryl esters to total lipids ratio in small HDL (%)
Free cholesterol to total lipids ratio in small HDL (%)
Triglycerides to total lipids ratio in small HDL (%)
−10
−5
0
5
9
Beta/SE

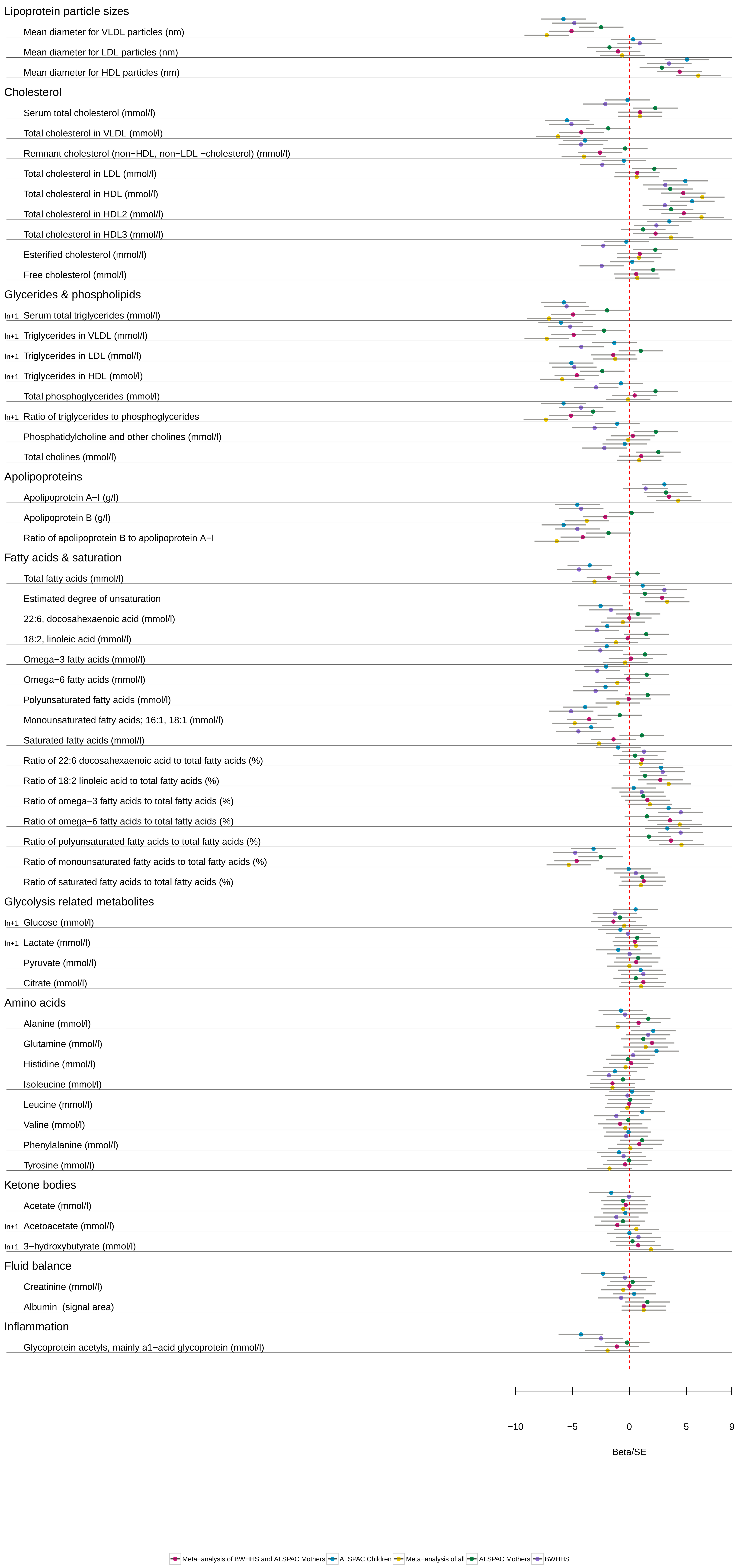

Lipoprotein particle sizes
Mean diameter for VLDL particles (nm)
Mean diameter for LDL particles (nm)
Mean diameter for HDL particles (nm)
Cholesterol
Serum total cholesterol (mmol/l)
Total cholesterol in VLDL (mmol/l)
Remnant cholesterol (non−HDL, non−LDL −cholesterol) (mmol/l)
Total cholesterol in LDL (mmol/l)
Total cholesterol in HDL (mmol/l)
Total cholesterol in HDL2 (mmol/l)
Total cholesterol in HDL3 (mmol/l)
Esterified cholesterol (mmol/l)
Free cholesterol (mmol/l)
Glycerides & phospholipids
ln+1 Serum total triglycerides (mmol/l)
ln+1 Triglycerides in VLDL (mmol/l)
ln+1 Triglycerides in LDL (mmol/l)
ln+1 Triglycerides in HDL (mmol/l)
Total phosphoglycerides (mmol/l)
ln+1 Ratio of triglycerides to phosphoglycerides
Phosphatidylcholine and other cholines (mmol/l)
Total cholines (mmol/l)
Apolipoproteins
Apolipoprotein A−I (g/l)
Apolipoprotein B (g/l)
Ratio of apolipoprotein B to apolipoprotein A−I
Fatty acids & saturation
Total fatty acids (mmol/l)
Estimated degree of unsaturation
22:6, docosahexaenoic acid (mmol/l)
18:2, linoleic acid (mmol/l)
Omega−3 fatty acids (mmol/l)
Omega−6 fatty acids (mmol/l)
Polyunsaturated fatty acids (mmol/l)
Monounsaturated fatty acids; 16:1, 18:1 (mmol/l)
Saturated fatty acids (mmol/l)
Ratio of 22:6 docosahexaenoic acid to total fatty acids (%)
Ratio of 18:2 linoleic acid to total fatty acids (%)
Ratio of omega−3 fatty acids to total fatty acids (%)
Ratio of omega−6 fatty acids to total fatty acids (%)
Ratio of polyunsaturated fatty acids to total fatty acids (%)
Ratio of monounsaturated fatty acids to total fatty acids (%)
Ratio of saturated fatty acids to total fatty acids (%)
Glycolysis related metabolites
ln+1 Glucose (mmol/l)
ln+1 Lactate (mmol/l)
Pyruvate (mmol/l)
Citrate (mmol/l)
Amino acids
Alanine (mmol/l)
Glutamine (mmol/l)
Histidine (mmol/l)
Isoleucine (mmol/l)
Leucine (mmol/l)
Valine (mmol/l)
Phenylalanine (mmol/l)
Tyrosine (mmol/l)
Ketone bodies
Acetate (mmol/l)
ln+1 Acetoacetate (mmol/l)
ln+1 3−hydroxybutyrate (mmol/l)
Fluid balance
Creatinine (mmol/l)
Albumin (signal area)
Inflammation
Glycoprotein acetyls, mainly a1−acid glycoprotein (mmol/l)
−10
−5
0
5
9
Beta/SE
Meta−analysis of BWHHS and ALSPAC Mothers
ALSPAC Children
Meta−analysis of all
ALSPAC Mothers
BWHHS
