# Supplementary material for: Metabolic Characterization of a Rare Genetic Variation Within APOC3 and Its Lipoprotein Lipase–Independent Effects
Source: Circ Cardiovasc Genet. 2016 Jun 21;9(3):231–9. doi: 10.1161/CIRCGENETICS.115.001302 (PMC4920206; doi:10.1161/CIRCGENETICS.115.001302)
Supplement: Supplementary file 3 [file hcg-9-231-s003.pdf]

DOI: 10.1161/CIRCGENETICS.115.001302

Metabolic Characterization of a Rare Genetic Variation within *APOC3* and its Lipoprotein Lipase Independent Effects

Running title: *Drenos et al.; APOC3 metabolomics*

Fotios Drenos, PhD[1,2]; George Davey Smith, MD, DSc[1]; Mika Ala-Korpela, PhD[1,3,4]; Johannes Kettunen, PhD[3-5]; Peter Würtz, PhD[3]; Pasi Soininen, PhD[3, 4]; Antti J. Kangas, MSc[3]; Caroline Dale, PhD[6]; Debbie A. Lawlor, MBChB, PhD[1]; Tom R. Gaunt, PhD[1]; Juan-Pablo Casas, PhD[2, 6]; Nicolas J. Timpson, PhD[1]

[1]MRC Integrative Epidemiology Unit, School of Social and Community Medicine, University of Bristol, Bristol; [2]Institute of Cardiovascular Science, University College London, London, United Kingdom; [3]Computational Medicine, Faculty of Medicine, University of Oulu & Biocenter Oulu, Oulu; [4]NMR Metabolomics Laboratory, School of Pharmacy, University of Eastern Finland, Kuopio; [5]Public Health Genomics Unit, Department of Chronic Disease Prevention, National Institute for Health and Welfare, Helsinki, Finland; [6]Department of Non-communicable Disease Epidemiology, London School of Hygiene and Tropical Medicine, London, United Kingdom

Circulation: Cardiovascular Genetics
American Heart Association.

Correspondence:
Nicolas J. Timpson, PhD
MRC Integrative Epidemiology Unit
University of Bristol
Oakfield House, Oakfield Grove
Bristol BS8 2BN
Tel: +44 117 3310131
E-mail: n.j.timpson@bris.ac.uk

Journal Subject Terms: Metabolism; Genetics; Genetic, Association Studies; Lipids and Cholesterol

Abstract:

Background - Plasma triglyceride levels (TG) have been implicated in atherosclerosis and coronary heart disease (CHD). Apolipoprotein C-III (APOC3) plays a key role in the hydrolysis of triglyceride rich lipoproteins to remnant particles by lipoprotein lipase (LPL) and their uptake by the liver. A rare variant in *APOC3*(rs138326449) has been associated with TG, very-low density lipoprotein (VLDL) and high density lipoprotein (HDL) levels, as well as risk of CHD. We aimed to characterize the impact of this locus across a broad set of mainly lipids focused metabolic measures.

Methods and Results - A high-throughput serum nuclear magnetic resonance metabolomics platform was used to quantify 225 metabolic measures in 13,285 participants from two European population cohorts. We analysed the effect of the *APOC3* variant on the metabolic measures and used the common *LPL*(rs12678919) polymorphism to test for LPL independent effects. 142 metabolic measures showed evidence of association with *APOC3*(rs138326449). In addition to previously reported TG and HDL associations, the variant was also associated with VLDL and HDL composition measures, other cholesterol measures and fatty acids. Comparison of the *APOC3* and *LPL* associations revealed that measures of medium and very large VLDL composition could not be predicted by the action of APOC3 through LPL.

Conclusions - We characterized the effects of the rare *APOC3*(rs138326449) loss of function mutation in lipoprotein metabolism, as well as the effects of *LPL*(rs12678919). Our results improve our understanding of the role of *APOC3* in TG metabolism, its *LPL* independent action, and the complex and correlated nature of human metabolites.

American Heart Association. Circulation Cardiovascular Genetics

Key words: metabolism; lipids; triglycerides; genetics; genetics, association studies; APOC3; LPL; VLDL; ALSPAC; BWHHS

High triglycerides (TG) levels have been consistently linked to risk of cardiovascular disease (CVD) [1], with mounting evidence supporting their causal role in the progression of the disease [2-5]. In moderately raised concentrations (2-10 mml/L) [6], TG appear to be able to penetrate the arterial intima [7] where they are trapped within the arterial wall contributing to atherosclerosis [8]. In the fasting state, circulating TG are transported in large, medium and small particles of very-low density lipoproteins (VLDLs) and their remnants after lipolysis and remodelling [9], mainly in the form of very small VLDLs and intermediate density lipoproteins (IDL). Smaller percentages of TG can also be found in LDL (<10%) and HDL (~ 15%) particles [10]. In postprandial conditions, chylomicrons and their remnants account for a large proportion of the elevated TG levels [11] .

American Heart Association. Circulation Cardiovascular Genetics

A common component of TG rich lipoproteins (TRLs) is apolipoprotein C3 (APOC3). APOC3 is a small 99-amino-acid peptide [12] coded by the *APOC3* gene located on chromosome 11, between and in close proximity to the *APOA4* and *APOA1* genes [13]. Recent advances in genetic data collection have permitted the study of low minor allele frequency variants (<5% MAF) which have, potentially, strong associations with phenotypes. Through these methods, a rare loss of function single nucleotide variant (SNV) rs138326449 that changes the splicing of the *APOC3* gene [10, 14-16] has been identified. The rare allele of this SNV has been associated with a substantial decrease in the risk of coronary artery disease (CAD) [10, 15], varying levels of reduction in TG of 0.5 to 1.5mmol/L depending on the population studied, and evidence of changes to VLDL and HDL levels [16].

APOC3 is involved in a number of intra- and extra- cellular mechanisms including the production and clearance of TRLs from circulation. The effect of APOC3 on TG and remnant particles, smaller and denser remodelled TG rich particles with some of their TG removed, is

suggested to operate mainly though the inhibition of TRLs hydrolysis by LPL [17] and a subsequent attenuation of uptake into hepatocytes [12]. *LPL* polymorphisms have been associated with levels of both TG and HDL [18], and also with risk of CAD [19]. To date, epidemiological studies have not had the required information to assess the molecular mechanisms involved, and despite the consensus that APOC3 is affecting the TLRs, small *in-vivo* and *in-vitro* studies have not determined the relative importance of the different mechanisms [20] and how specific mutations of the *APOC3* gene can affect these processes [21].

Here we use data from two well characterised European population cohort studies to provide a detailed profile of the associations between the rare and poorly characterised *APOC3* genetic variant rs138326449 and individual lipoprotein subclasses that might contribute to atherosclerosis risk. For this we used a targeted metabolomics approach measuring, among others, the size and composition of 14 lipoprotein subclasses. This approach has previously been used to characterise the molecular profile of common diseases, identify new biomarkers, and study the genetic basis of systemic metabolism (reviewed in Soininen et al 2015 [22]). We aimed to characterise in greater detail the impact of variation at this locus and its role in TG metabolism as seen from an epidemiological perspective, including elucidating APOC3's LPL-dependent and LPL-independent actions on the levels and composition of specific lipoprotein particles as well as the mechanism of action of a recently proposed *APOC3* inhibitor for the treatment of hypertriglyceridemia.

American Heart Association. Circulation Cardiovascular Genetics

Methods

Study populations

The Avon Longitudinal Study of Parents and Children (ALSPAC) is a population based, prospective birth cohort (www.bris.ac.uk/alspac). The study initially invited >14000 pregnancies

and has since followed participants in a number of phases during development and maturity. Information on the phases can be found at www.bris.ac.uk/alspac/researchers/resources-available/data-details/data-tables/documents/focusclinicsessions.pdf . Full details of the study have been published previously and here focus is on the offspring of this study (herein referred to as young participants) and their mothers [23]. Ethical approval for the study was obtained from the ALSPAC Ethics and Law Committee and from the UK NHS National Health Service Local Research Ethics Committees. Participants have provided informed consent for the use of the data.

Analyses were also undertaken in an independent cohort of women. The British Women Heart Health Study (BWHHS), is a prospective cohort study that recruited women between the ages of 60 and 79 years from 23 towns across the United Kingdom (UK) between 1999–2001 and has followed those women forward through record linkage and detailed questionnaires since that time [24]. Ethical approval for the study was obtained from the UK NHS Research Ethics Committees and participants provided informed consent.

American Heart Association. Circulation Cardiovascular Genetics

Serum NMR metabolomics

A high-throughput serum nuclear magnetic resonance (NMR) metabolomics platform was used to quantify up to 233 metabolic measures that represent a broad molecular signature of systemic metabolism [22, 25]. The measured set covers multiple metabolic pathways, including lipoprotein lipids and subclasses, fatty acids and fatty acid compositions, as well as amino acids and glycolysis precursors. All molecular measures are quantified in a single experimental setup, constituting both established and novel metabolic risk factors [22]. The applied NMR-based metabolic profiling has recently been used in various epidemiological and genetic studies [26-31]. Applications of this high-throughput metabolomics platform has recently been reviewed [22] and

details of the experimentation have been described elsewhere [25, 32] .

For the ALSPAC young participants, metabolic measures were obtained from serum taken at follow-up clinic assessments at the approximate ages of 7, 15 and 17 years. In total 7,176 participants had at least one measurement, with 1,453 measured at all three ages. For 73 sibling pairs, one child was removed at random before statistical analysis. Samples from the 15 and 17 year follow-up assessments were taken after overnight fast, for those assessed in the morning, and at least 6-hours fast for those assessed after 14.00; samples taken at the 7-year assessment were non-fasted. To allow the maximum sample size for analyses (given the relative stability of non-fasting versus non-fasting samples [33]), data were taken from all available time points. However, to minimise unnecessary heterogeneity, where participants had repeat measurements, we prioritised those collected under fasted conditions. This led to a final analysis sample with 37.5% non-fasting (sensitivity analyses excluding those non-fasting are provided in the supplementary material).

American Heart Association. Circulation Cardiovascular Genetics

Measurements were available for 4,530 ALSPAC mothers at a median age of 48 years (with an overlap of 1,981 mothers of the young participant sample). All of the samples from the ALSPAC mothers taken were after an overnight fast for those taken in the morning and a minimum 6-hours for those taken after 14.00.

Samples were available for 3,780 women from the BWHHS at baseline assessment (age 60-79 years) after an overnight fast for those assessed in the morning and a minimum of 6-hours for those assessed after 14.00. Due to differences in storage of the samples between studies, 225 metabolites were common in all three and are used here.

Genotyping

Genotyping for the rs138326449 splice variant *APOC3* mutation was performed using KASPAR

at KBioscience (www.lgcgenomics.com) for all participants from both studies who had a suitable DNA sample. Genotypes for the leading TG associated *LPL* singe nucleotide polymorphism (SNP) rs12678919, a downstream intergenic variant in linkage disequilibrium with a SNP with previous evidence of transcriptional regulation [34], were extracted from the existing genome wide common variant data available in ALSPAC [35]. For BWHHS, the SNP was extracted from the available Metabochip array data [36]. Standard metrics were employed to assess the quality of these data (missingness (>3%), non-European ancestry, and SNPs of minor allele frequency of <1%, call rate of <95%, and Hardy–Weinberg equilibrium (HWE) ($p<5\times10^{-7}$))

Statistical analysis

American Heart Association. Circulation Cardiovascular Genetics

The metabolic measures were inspected for deviations from normality and transformed, when needed, using the natural logarithm plus one in order to be consistent throughout since some metabolic measures included zero values. For the analysis of the association between the metabolic measures and rs138326449(*APOC3*) we used a linear regression model adjusting for age, sex and, in order to try to adjust for the non-fasting measurements at age 7, as well as any other differences related to handling and storage of the blood samples, an indicator variable for the phase of measurement where relevant. Primary analysis was undertaken in the ALSPAC young participants. The meta-analysed results, from a fixed effects model, of ALSPAC mothers and BWHHS were generated in parallel to those from the ALSAPC offspring data since the differences between them permit the confirmation of the common observed associations, or lack of, but hinder the distinction between differences due to false positives or due to fasting status, age and sex for the discordant results. For the overall meta-analysis of the three samples we first pooled the ALSPAC mothers and young participants using a linear mixed effects model to adjust for the pedigree correlation [37]. Subsequently we used a fixed effect meta-analysis to combine the

results of the pooled ALSPAC sample with the BWHHS women. To address the uncertainty due to heterogeneity, we also used a random effects meta-analysis model, but due to the small number of samples available we consider this as a sensitivity analysis of the main fixed effects results[38]. We used the Benjamini and Yekutieli false discovery rate (FDR) procedure under dependency [39] to adjust the p-values of the confirmation analysis for the associations reaching the 0.05 p-value threshold in the discovery sample and all meta-analysis results for multiple testing as implemented in the p.adjust package in R.

It is assumed that the APOC3 acts on TG and VLDL largely though inhibition of LPL [12, 17]. We tested whether the *APOC3* variant associations with metabolites could be explained by the inhibition of LPL by using the genetic variant *LPL*(rs12678919) as a proxy of LPL protein levels [18]. We estimated the predicted effect of *APOC3* on the metabolites if this was exclusively through LPL by looking at the ratio of SNP associations between *LPL*(rs12678919) and the focus variant here *APOC3*(rs138326449). The model used the mean of the ratios of the effects of *APOC3* and *LPL* genetic variants on each metabolite to obtain an estimate of the LPL mediated effects of APOC3. To avoid the inclusion of metabolic measures that do not follow the assumed model of APOC3 action, we made use of the 25% trimmed mean as a true estimate of the effect of APOC3 on LPL. We bootstrapped the sample 1,000 times to obtain the standard error of the mean of the ratios. The predicted effect of *APOC3* on each metabolite was estimated as the product of the mean of the ratios and the *LPL* effect per metabolite, while the predicted confidence intervals were estimated taking into account the standard errors of both the *LPL* estimates and of the mean of the ratios. The absence of overlap in the coefficients between the predicted and observed *APOC3* estimates were considered as evidence for an effect of APOC3 not mediated by its inhibition of LPL. We followed the same procedure in each of the parallel

American Heart Association. Circulation Cardiovascular Genetics

studies and combined their estimates using a fixed effects meta-analysis weighted by their sample size.

Analyses were undertaken in Stata (Stata Statistical Software: Release 13. College Station, TX: StataCorp LP) and R 3.1.0 [40] and plots prepared using ggplot2 [41].

Results

APOC3(rs138326449) was present with minor allele frequencies (MAF) of 0.20 to 0.28% in the three studies and adhered to HWE. *LPL*(rs12678919) polymorphism was more common with a MAF of 9.2 to 10.6% across the three studies. Key characteristic of participants from each study are shown in Table 1.

Of the 225 metabolic measures available in all three studies, when analysed in the ALSPAC young participants, 134 showed nominal evidence of association with *APOC3*(rs138326449) ($p \leq 0.05$). *APOC3*(rs138326449) was associated with a decrease in TG concentration (-0.11 mmol/L of geometric mean, 95%CI -0.16 to -0.05 mmol/L, $p = 2.57 \times 10^{-4}$) and an increase of HDL (0.26 mmol/L, 95%CI 0.18 to 0.34 mmol/L, $p = 2.4 \times 10^{-6}$). More generally *APOC3*(rs138326449) showed an effect on a broad range of measures reflecting the circulating levels and lipid composition of VLDL and HDL particles (Figure 1). A full table of results for all 225 measures can be found in the supplementary materials as Table S1. A comparison of the results from the mixed fasting-non-fasting analysis used and analysis based on fasting and non-fasting measurements showed broadly similar association profiles, though there were differences in the non-fasting samples for specific measures of small HDL, very large VLD, large LDL, remnant cholesterol, fatty acids and glutamine (Table S2).

In meta-analysis results of the ALSPAC mothers and BWHHS samples, 124 metabolic measures showed nominal evidence of association with *APOC3*(rs138326449) ($p \leq 0.05$) (Table

American Heart Association. Circulation Cardiovascular Genetics

S1), supporting 81 of the signals seen in the ALSPAC younger participants after adjustment for multiple testing (Table S3). For both TG and HDL, associations were stronger, but consistent with the young participant results (-0.23 mmol/L of geometric mean, 95% CI -0.34 to -0.12 mmol/L, $p = 4.6\times10^{-6}$ and 0.4191 mmol/L, 95% CI 0.28 to 0.56 mmol/L, $p = 1.74\times10^{-9}$ respectively). In addition to the VLDL and HDL measures of concentration and composition, evidence for an increase of the ratio of ω-6, polyunsaturated and monounsaturated fatty acids to total fatty acid were also confirmed. When all three samples were considered together 118 associations were observed after adjustment for multiple testing, with 88 also showing evidence of association in the random effects model. Associations in ALSPAC young participants, meta-analysis of ALSPAC Mothers and BWHHS and the meta-analysis of all three samples are presented in Figure S1 with details for each association in Table S1.

American Heart Association. Circulation Cardiovascular Genetics

The pattern of associations between *LPL* (rs12678919) and the metabolic measures was very similar to that observed for *APOC3* (rs138326449) (Figure 2). A Pearson's correlation test between the coefficients of *LPL* (rs12678919), scaled by their SE, and those obtained for the association of the metabolites with *APOC3* (rs138326449) in the ALSPAC young participants shows strong correlation with r equal to 0.88. In ALSPAC young participants, a total of 126 metabolic measures showed nominal evidence of association with *LPL* (rs12678919) ($p \leq 0.05$) (Table S4). Of these associations, 90 were confirmed in the meta-analysis of ALSPAC mothers and BWHHS samples (FDR adjusted $p \leq 0.05$) (Table S5). The meta-analysis of all three samples revealed 113 associations (FDR adjusted $p \leq 0.05$), with 75 of them having evidence of association when a mixed effects model was considered. All association results for the analyses can be seen in Table S4 and plotted against the discovery effects in Figure S2.

There was evidence for *APOC3* effects being mediated through LPL in the majority of

the metabolic measures considered. Of the 225 measures tested in the ALSPAC young participants, six had no overlapping 95% confidence intervals between the directly observed *APOC3*(rs138326449) effects and those predicted by *LPL*(rs12678919) (Table S6). The ALSPAC Mothers and the BWHHS results confirmed two of the suggested six APOC3 specific effects as independent from LPL (Table 2 and Figure 3). When all three samples where combined eight measures showed evidence of non-overlapping estimates (Table S6). Those measures which maintained evidence of an effect outside the action of LPL inhibition were both measures of VLDL composition characterising the percentage of TG in very large and medium VLDL. All results on the comparison of the LPL predicted and observed APOC3 effects are provided in Table S6.

American Heart Association. Circulation Cardiovascular Genetics

Discussion

Using detailed measures of lipoprotein subclass concentration and composition and a number of other metabolic measures we provided a profile for the effect of the rare *APOC3* (rs138326449) splice variant on mothers and young participants from one European population cohort and older adult women from an independent European population cohort. We confirmed the previously reported, but crudely assessed, association with TG, VLDL and HDL [16] levels and identified additional associations with VLDL and HDL composition, other cholesterol measures and fatty acids. Using *LPL* (rs12678919), as a proxy for LPL protein levels, we tested the extent to which APOC3 action on lipids is mediated through LPL inhibition[12, 17]. Our results suggest that the great majority of the APOC3 effects are in line with the assumed mode of action through LPL, the composition of very large and medium VLDL particles involve other mechanisms in addition to LPL. Although the pathways involved have been previously studied in model organisms and *in-vitro* experiments [21], here we present an epidemiological view of TG metabolism in relation to

APOC3 based on extensive metabolite measurements, many of which have not been previously studied in large epidemiological studies. Biological interpretation of the identified associations should thus be in the context of previously accumulated evidence in other experimental systems many of which we were able to replicate here as also operating in humans.

Although the associations with total VLDL, HDL and TG levels were in the same direction as those in previous studies [10, 15, 16, 42], their magnitude of effect was lower in the young participants, probably due to the shorter exposure time to the action of the mutation. We did not find evidence for the associations of *APOC3* (rs138326449) with either IDL or LDL concentration or composition, with previous studies reporting contradictory effects on LDL for the rare *APOC3* mutations [15] and its mRNA inhibition [43]. Consideration of the fasting and non-fasting individuals in the young participants sample separately was consistent with the overall results of no association.

The greater resolution provided by the detail measurement of the lipoprotein subclasses shows that the effects of the splice variant on VLDL and HDL can be seen in almost the entire spectrum of their size. A highly similar pattern can be seen in the *LPL* associations with the metabolic measures. Also, both have an effect on the diameter of VLDL and HDL particles, with the rare allele associated with a decrease in the diameter of VLDL and an increase in the diameter of HDL particles in serum. For VLDL, lipoprotein kinetic studies have shown that the different size VLDL particles are metabolically heterogeneous [44], with large subfractions generating remnants that persist in circulation, while smaller VLDL particles are rapidly and efficiently converted to LDL [44], which agrees with our observations especially for the very small VLDL and LDL measures.

Although the assumed mode of effect of APOC3 on TG levels and TRLs is due to

American Heart Association. Circulation Cardiovascular Genetics

impeded lipolytic conversion and hepatic clearance, in-vivo and in-vitro evidence point towards an additional role of APOC3 in the production of high TG content VLDL (reviewed in Yao and Wang [21]). Our comparison of the *APOC3* and *LPL* association revealed that the composition of medium and very large VLDL is not fully predicted by the action of APOC3 through LPL. Studies show that *APOC3* expression promotes the assembly and secretion of the bigger TG rich VLDL from hepatocytes through the mobilisation of endoplasmic reticulum/Golgi microsomes triglyceride for VLDL assembly[45]. This intracellular mechanism becomes manifest under conditions of insulin resistance or hypertriglyceridemia [21], though our results suggest it also operates under normal conditions. Different structural changes in the APOC3 protein, either in the N or C terminals, affect the assembly and secretion of larger VLDL particles in different ways [46 47] but have no effect on the TG poor smaller VLDL particles.

American Heart Association. Circulation Cardiovascular Genetics

Our conclusions of an APOC3 LPL-independent TG related pathway agree with Gaudet et al [48] testing the effects of an *APOC3* mRNA inhibitor on familial chylomicronemia syndrome sufferers. In this case, deficiency in LPL leads to severe hypertriglyceridemia which can result in recurrent and potentially fatal pancreatitis. When three patients were given an *APOC3* inhibitor that lowered their APOC3 levels, a reduction of TG was observed [48]. Similar results were obtained in patients with severe or uncontrolled hypertriglyceridemia [43]. Although we did not find an LPL-independent effect on total serum TG, either due to lack of statistical power or due to the differences between hypertriglyceridemia patients and the samples available here representing the general population, the availability of more refined measures of TG concentration in specific subclasses permitted the identification of the likely mechanism responsible for the effect of APOC3 inhibition. Our results point towards changes in the composition of VLDL and its proportion of TG though an intrahepatic pathway, rather than a

mechanism involving changes in triglyceride absorption.

Our study has a number of limitations, mainly in relation to the differences in age and sex between the three study samples and the mix of fasting and non-fasting status in ALSPAC children. For these reasons, the ALSPAC mothers and BWHHS samples were only considered as able to confirm the common observed associations, with false positives indinguishable from heterogeneity between the samples due to age and sex for the discordant result. Fasting status in the ALSPAC young participants was addressed through a sensitivity analysis excluding non-fasting individuals, with no evidence of an effect that can change our conclusions found. Finally, the low number of the rare APOC3(rs138326449) variant carriers might have contributed to the no identification of true associations, due to low statistical power, especially in the proportional modelling part of our work.

American Heart Association. Circulation Cardiovascular Genetics

To summarise, we were able to refine and characterise the effects of the newly discovered *APOC3*(rs138326449) loss of function mutation in lipoprotein metabolism and its potential to affect TG levels. We also characterised the effects of the GWAS lead signal in the area of *LPL* rs12678919 and compared its action to that of the APOC3 variant. Our findings suggest that the APOC3 variant has a wide range of actions on lipids and fatty acids beyond its known effect on TG and HDL. Whilst our novel analyses suggest that much of the action of APOC3 on lipids is mediated via LPL action, as hypothesised, a parallel intracellular mechanism previously only observed in model organisms and cell cultures under conditions mimicking pathophysiological disorders also appear to be relevant for the composition of VLDL particles. Our results support the results of clinical trials on LPL deficient patients for ISIS 304801, an antisense oligonucleotide inhibitor of *APOC3* mRNA and thus illustrate the possible use of such approaches as a relatively quick and low cost tool in the evaluation of drug targets.

Acknowledgments: We are extremely grateful to all the families who took part in this study, the midwives for their help in recruiting them, and the whole ALSPAC team, which includes interviewers, computer and laboratory technicians, clerical workers, research scientists, volunteers, managers, receptionists and nurses. This publication is the work of the authors and FD and NJT will serve as guarantors for the contents of this paper. GWAS data was generated by Sample Logistics and Genotyping Facilities at the Wellcome Trust Sanger Institute and LabCorp (Laboratory Corporation of America) using support from 23andMe.

Funding Sources: The UK Medical Research Council and the Wellcome Trust (Grant ref: 102215/2/13/2) and the University of Bristol provide core support for ALSPAC. Grants from the British Heart Foundation (SP/07/008/24066) and Wellcome Trust (WT092830M and WT088806) funded data collection from the ALSPAC mothers. The British Women's Heart and Health Study has been supported by funding from the British Heart Foundation (BHF) (grant PG/13/66/304422). FD, NJT, GDS, DAL all work in a Unit receiving funds from the UK Medical Research Council (MC_UU_12013/1-9). DAL is a UK NIH Research Senior Investigator (NF-SI-0611-10196). PW is funded by the Finnish Diabetes Research Foundation and the Novo Nordisk Foundation. JK was supported from the Academy of Finland (grant number 283045). The quantitative serum NMR metabolomics platform and its development have been supported by the Academy of Finland, TEKES (the Finnish Funding Agency for Technology and Innovation), the Sigrid Juselius Foundation, the Novo Nordisk Foundation, the Finnish Diabetes Research Foundation, the Paavo Nurmi Foundation, and the strategic and infrastructural research funding from the University of Oulu, Finland, as well as by the British Heart Foundation, the Wellcome Trust and the Medical Research Council, UK. The views expressed in this paper are those of the authors and not necessarily any funding body. The funders did not have any influence over data collection, analyses, and interpretation of findings or writing of this paper.

Disclosures: A.J.K., P.S., P.W., J.K. and M.A.K. are shareholders of Brainshake Ltd (www.brainshake.fi), a company offering NMR-based metabolite profiling. A.J.K., P.S., P.W. and J.K. report employment and consulting for Brainshake Ltd. No relevant conflicts of interest by other authors.

American Heart Association. Circulation Cardiovascular Genetics

References:

1. Nordestgaard BG, Benn M, Schnohr P, Tybjærg-Hansen A. Nonfasting triglycerides and risk of myocardial infarction, ischemic heart disease, and death in men and women. *JAMA*. 2007;298:299-308.

2. Thomsen M, Varbo A, Tybjaerg-Hansen A, Nordestgaard BG. Low Nonfasting Triglycerides and Reduced All-Cause Mortality: A Mendelian Randomization Study. *Clin Chem*. 2014;60:737-746.

3. Varbo A, Benn M, Tybjaerg-Hansen A, Jorgensen AB, Frikke-Schmidt R, Nordestgaard BG. Remnant Cholesterol as a Causal Risk Factor for Ischemic Heart Disease. *J Am Coll Cardiol*. 2013;61:427-436.

4. Goldberg IJ, Eckel RH, McPherson R. Triglycerides and Heart Disease Still a Hypothesis? *Arterioscler Thromb Vasc Biol*. 2011;31:1716-1725.

5. Holmes MV, Asselbergs FW, Palmer TM, Drenos F, Lanktree MB, Nelson CP, et al. Mendelian randomization of blood lipids for coronary heart disease. *Eur Heart J*. 2015;36:539-550.

6. Nordestgaard BG, Varbo A. Triglycerides and cardiovascular disease. *Lancet*. 2014;384:626-635.

7. Shaikh M, Wootton R, Nordestgaard BG, Baskerville P, Lumley JS, La Ville AE, et al. Quantitative studies of transfer in vivo of low density, Sf 12-60, and Sf 60-400 lipoproteins between plasma and arterial intima in humans. *Arterioscler Thromb Vasc Biol*. 1991;11:569-577.

8. Rutledge JC, Mullick AE, Gardner G, Goldberg IJ. Direct visualization of lipid deposition and reverse lipid transport in a perfused artery - Roles of VLDL and HDL. *Circ Res*. 2000;86:768-773.

9. Chapman MJ, Ginsberg HN, Amarenco P, Andreotti F, Borén J, Catapano AL, et al. Triglyceride-rich lipoproteins and high-density lipoprotein cholesterol in patients at high risk of cardiovascular disease: evidence and guidance for management. *Eur Heart J*. 2011;32:1345-1361.

10. Jorgensen AB, Frikke-Schmidt R, Nordestgaard BG, Tybjaerg-Hansen A. Loss-of-function mutations in APOC3 and risk of ischemic vascular disease. *N Engl J Med*. 2014;371:32-41.

11. Xiao C, Lewis GF. Regulation of chylomicron production in humans. *Biochim Biophys Acta*. 2012;1821:736-746.

12. Ooi EM, Barrett PH, Chan DC, Watts GF. Apolipoprotein C-III: understanding an emerging cardiovascular risk factor. *Clin Sci*. 2008;114:611-624.

American Heart Association
Circulation
Cardiovascular Genetics

13. Kent WJ, Sugnet CW, Furey TS, Roskin KM, Pringle TH, Zahler AM, et al. The Human Genome Browser at UCSC. *Genome Res*. 2002;12:996-1006.

14. Tachmazidou I, Dedoussis G, Southam L, Farmaki A-E, Ritchie GRS, Xifara DK, et al. A rare functional cardioprotective APOC3 variant has risen in frequency in distinct population isolates. *Nat Commun*. 2013;4:2872.

15. Crosby J, Peloso GM, Auer PL, Crosslin DR, Stitziel NO, Lange LA, et al. Loss-of-function mutations in APOC3, triglycerides, and coronary disease. *N Engl J Med*. 2014;371:22-31.

16. Timpson NJ, Walter K, Min JL, Tachmazidou I, Malerba G, Shin S-Y, et al. A rare variant in APOC3 is associated with plasma triglyceride and VLDL levels in Europeans. *Nat Commun*. 2014;5:4871.

17. Ginsberg HN, Le NA, Goldberg IJ, Gibson JC, Rubinstein A, Wang-Iverson P, et al. Apolipoprotein B metabolism in subjects with deficiency of apolipoproteins CIII and AI. Evidence that apolipoprotein CIII inhibits catabolism of triglyceride-rich lipoproteins by lipoprotein lipase in vivo. *J Clin Invest*. 1986;78:1287-1295.

18. Global Lipids Genetics C. Discovery and refinement of loci associated with lipid levels. *Nat Genet*. 2013;45:1274-1283.

19. Dichgans M, Malik R, König IR, Rosand J, Clarke R, Gretarsdottir S, et al. Shared Genetic Susceptibility to Ischemic Stroke and Coronary Artery Disease: A Genome-Wide Analysis of Common Variants. *Stroke*. 2014;45:24-36.

20. Zheng C, Khoo C, Ikewaki K, Sacks FM. Rapid turnover of apolipoprotein C-III-containing triglyceride-rich lipoproteins contributing to the formation of LDL subfractions. *J Lipid Res*. 2007;48:1190-1203.

21. Yao Z, Wang Y. Apolipoprotein C-III and hepatic triglyceride-rich lipoprotein production. *Curr Opin Lipidol*. 2012;23:206-212.

22. Soininen P, Kangas AJ, Würtz P, Suna T, Ala-Korpela M. Quantitative Serum Nuclear Magnetic Resonance Metabolomics in Cardiovascular Epidemiology and Genetics. *Circ Cardiovasc Genet*. 2015;8:192-206.

23. Boyd A, Golding J, Macleod J, Lawlor DA, Fraser A, Henderson J, et al. Cohort Profile: the 'children of the 90s'--the index offspring of the Avon Longitudinal Study of Parents and Children. *Int J Epidemiol*. 2013;42:111-127.

24. Lawlor DA, Bedford C, Taylor M, Ebrahim S. Geographical variation in cardiovascular disease, risk factors, and their control in older women: British Women's Heart and Health Study. *J Epidemiol Community Health*. 2003;57:134-140.

American Heart Association
Circulation
Cardiovascular Genetics

25. Soininen P, Kangas AJ, Wurtz P, Tukiainen T, Tynkkynen T, Laatikainen R, et al. High-throughput serum NMR metabonomics for cost-effective holistic studies on systemic metabolism. *Analyst*. 2009;134:1781-1785.

26. Würtz P, Mäkinen V-P, Soininen P, Kangas AJ, Tukiainen T, Kettunen J, et al. Metabolic Signatures of Insulin Resistance in 7,098 Young Adults. *Diabetes*. 2012;61:1372-1380.

27. Kettunen J, Tukiainen T, Sarin A-P, Ortega-Alonso A, Tikkanen E, Lyytikainen L-P, et al. Genome-wide association study identifies multiple loci influencing human serum metabolite levels. *Nat Genet*. 2012;44:269-276.

28. Kujala UM, Mäkinen V-P, Heinonen I, Soininen P, Kangas AJ, Leskinen TH, et al. Long-term Leisure-time Physical Activity and Serum Metabolome. *Circulation*. 2013;127:340-348.

29. Fischer K, Kettunen J, Würtz P, Haller T, Havulinna AS, Kangas AJ, et al. Biomarker Profiling by Nuclear Magnetic Resonance Spectroscopy for the Prediction of All-Cause Mortality: An Observational Study of 17,345 Persons. *PLoS Med*. 2014;11:e1001606.

30. Würtz P, Wang Q, Kangas AJ, Richmond RC, Skarp J, Tiainen M, et al. Metabolic Signatures of Adiposity in Young Adults: Mendelian Randomization Analysis and Effects of Weight Change. *PLoS Med*. 2014;11:e1001765.

31. Würtz P, Havulinna AS, Soininen P, Tynkkynen T, Prieto-Merino D, Tillin T, et al. Metabolite Profiling and Cardiovascular Event Risk: A Prospective Study of 3 Population-Based Cohorts. *Circulation*. 2015;131:774-785.

32. Inouye M, Kettunen J, Soininen P, Silander K, Ripatti S, Kumpula LS, et al. Metabonomic, transcriptomic, and genomic variation of a population cohort. *Mol Syst Biol*. 2010;6:441-441.

33. Sidhu D, Naugler C. Fasting time and lipid levels in a community-based population: A cross-sectional study. *Arch Intern Med*. 2012;172:1707-1710.

34. Smith AJP, Palmen J, Putt W, Talmud PJ, Humphries SE, Drenos F. Application of statistical and functional methodologies for the investigation of genetic determinants of coronary heart disease biomarkers: lipoprotein lipase genotype and plasma triglycerides as an exemplar. *Hum Mol Genet*. 2010;19:3936-3947.

35. Bonnelykke K, Matheson MC, Pers TH, Granell R, Strachan DP, Alves AC, et al. Meta-analysis of genome-wide association studies identifies ten loci influencing allergic sensitization. *Nat Genet*. 2013;45:902-906.

36. Shah T, Engmann J, Dale C, Shah S, White J, Giambartolomei C, et al. Population Genomics of Cardiometabolic Traits: Design of the University College London-London School of Hygiene and Tropical Medicine-Edinburgh-Bristol (UCLEB) Consortium. *PLoS One*. 2013;8:e71345.

American Heart Association. Circulation Cardiovascular Genetics

37. Chen M-H, Yang Q. GWAF: an R package for genome-wide association analyses with family data. *Bioinformatics*. 2010;26:580-581.

38. Higgins JPT, Thompson SG, Spiegelhalter DJ. A re-evaluation of random-effects meta-analysis. *J R Stat Soc Ser A Stat Soc*. 2009;172:137-159.

39. Benjamini Y, Yekutieli D. The Control of the False Discovery Rate in Multiple Testing under Dependency. *Ann Stat*. 2001;29:1165-1188.

40. R: A language and environment for statistical computing. [computer program]. Version 3.1.0. Vienna, Austria; 2014.

41. Wickham H. ggplot2: elegant graphics for data analysis. New York: Springer; 2009.

42. Graham MJ, Lee RG, Bell TA, Fu W, Mullick AE, Alexander VJ, et al. Antisense Oligonucleotide Inhibition of Apolipoprotein C-III Reduces Plasma Triglycerides in Rodents, Nonhuman Primates, and Humans. *Circ Res*. 2013;112:1479-1490.

43. Gaudet D, Alexander VJ, Baker BF, Brisson D, Tremblay K, Singleton W, et al. Antisense Inhibition of Apolipoprotein C-III in Patients with Hypertriglyceridemia. *N Engl J Med*. 2015;373:438-447.

44. Packard CJ, Shepherd J. Lipoprotein Heterogeneity and Apolipoprotein B Metabolism. *Arterioscler Thromb Vasc Biol*. 1997;17:3542-3556.

45. Sundaram M, Zhong S, Bou Khalil M, Links PH, Zhao Y, Iqbal J, et al. Expression of apolipoprotein C-III in McA-RH7777 cells enhances VLDL assembly and secretion under lipid-rich conditions. *J Lipid Res*. 2010;51:150-161.

46. Qin W, Sundaram M, Wang Y, Zhou H, Zhong S, Chang CC, et al. Missense mutation in APOC3 within the C-terminal lipid binding domain of human ApoC-III results in impaired assembly and secretion of triacylglycerol-rich very low density lipoproteins: evidence that ApoC-III plays a major role in the formation of lipid precursors within the microsomal lumen. *J Biol Chem*. 2011;286:27769-27780.

47. Sundaram M, Zhong SM, Khalil MB, Zhou H, Jiang ZG, Zhao Y, et al. Functional analysis of the missense APOC3 mutation Ala23Thr associated with human hypotriglyceridemia. *J Lipid Res*. 2010;51:1524-1534.

48. Gaudet D, Brisson D, Tremblay K, Alexander VJ, Singleton W, Hughes SG, et al. Targeting APOC3 in the Familial Chylomicronemia Syndrome. *N Engl J Med*. 2014;371:2200-2206.

American Heart Association
Circulation
Cardiovascular Genetics

DOI: 10.1161/CIRCGENETICS.115.001302

Table 1: Numbers of individuals measured and key characteristic of samples analysed

	ALSPAC Young participants	ALSPAC Mothers	BWHHS
N with NMR measurements	7176	4530	3646
N with NMR measurements and APOC3 information	6765	2463	3584
MAF of APOC3 %	0.20	0.26	0.28
N with NMR measurements and LPL information	5656	1953	1866
MAF of LPL	10.34	10.60	9.19
Sex (% females)	48.33	1	1
Age (years) (mean, interquartile range)	13.5 (7.5-17.7)	47.9 (45 - 51)	74.45 (70-79)
HDL mmol/L (mean , standard deviation)	1.44 (0.22)	1.69 (0.32)	1.67 (0.45)
TG mmol/L (geometric mean, untransformed standard deviation)	0.93 (0.18)	1.00 (0.24)	1.57 (0.31)

Circulation Cardiovascular Genetics American Heart Association

DOI: 10.1161/CIRCGENETICS.115.001302

Table 2: Observed and predicted effects of *APOC3* on metabolic measures with evidence of an LPL independent mechanism.

	Young participants								ALSPAC mothers and BWHHS							
	Observed				Predicted				Observed				Predicted			
	Beta coef.	SE	Upper 95% CI	Lower 95% CI	Beta coef.	SE	Upper 95% CI	Lower 95% CI	Beta coef.	SE	Upper 95% CI	Lower 95% CI	Beta coef.	SE	Upper 95% CI	Lower 95% CI
*Triglycerides to total lipids ratio in very large VLDL (%)	-0.297	0.062	-0.176	-0.419	-0.055	0.052	0.047	-0.156	-0.790	0.214	-0.371	-1.210	0.109	0.207	0.514	-0.295
*Triglycerides to total lipids ratio in medium VLDL (%)	-0.049	0.015	-0.019	-0.078	0.019	0.014	0.045	-0.008	-0.179	0.027	-0.126	-0.233	-0.021	0.027	0.031	-0.073

*Measures have been transformed using an ln+1 transformation.

Circulation Cardiovascular Genetics American Heart Association.

Figure Legends:

Figure 1: *APOC3*(rs138326449) associations with selected metabolic measures in plasma in ALSPAC young participants and BWHHS – ALSPAC mothers in Beta/SE units. The variant is associated predominately with VLDL and HDL concentration and composition as well as particle size, cholesterol measures and fatty acids. Multiple associations per particle are represented by a single entry. Information on the transformation used is also provided. Estimates and confidence intervals were scaled by the standard error of each measurement. Plot of the association of all 225 measured metabolites can be seen as Figure S1. A detailed list of effect sizes and p-values for all measures can be seen in Table S1.

American Heart Association
Circulation Cardiovascular Genetics

Figure 2: *APOC3*(rs138326449) and *LPL*(rs12678919) correlation of their respective associations with metabolic measures in plasma. The observed effects of *LPL*(rs12678919) are very similar to those seen with *APOC3*(rs138326449). The black line is the line of perfect fit while the blue line is the correlation between the two metabolic profiles of the two SNPs with slope equal to 0.87 for the ASPAC young participants. Estimates and confidence intervals were scaled by the standard error of each measurement. A detailed list of the association measures for all metabolites and the *LPL*(rs12678919) can be seen in Table S2 and plotted as Figure S2.

Figure 3: Expected and observed *APOC3* - metabolites associations for the subset of metabolites with an LPL-independent effect. The coefficients and CIs are scaled by the SE of the observed effect.

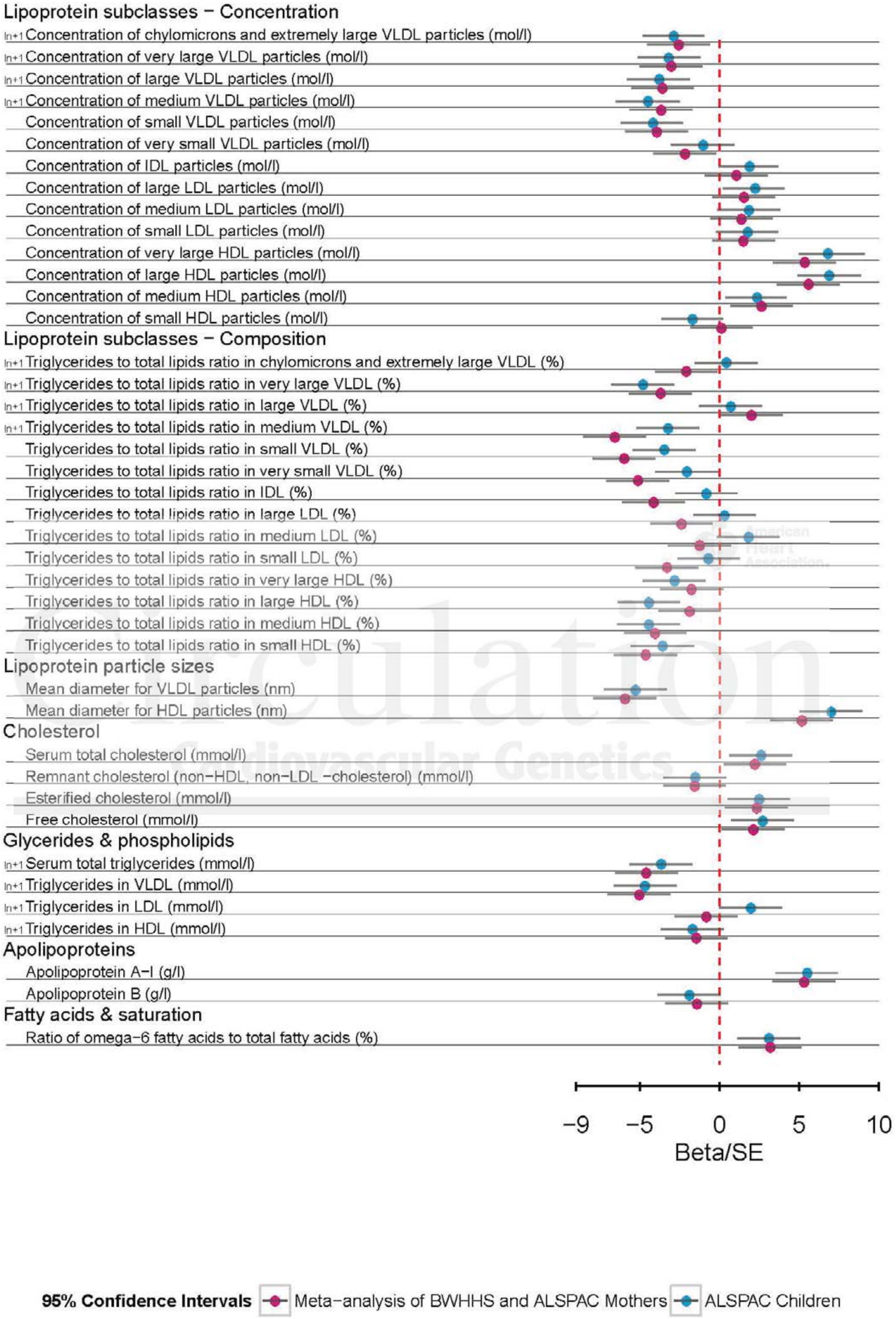

American Heart Association.
Circulation Cardiovascular Genetics

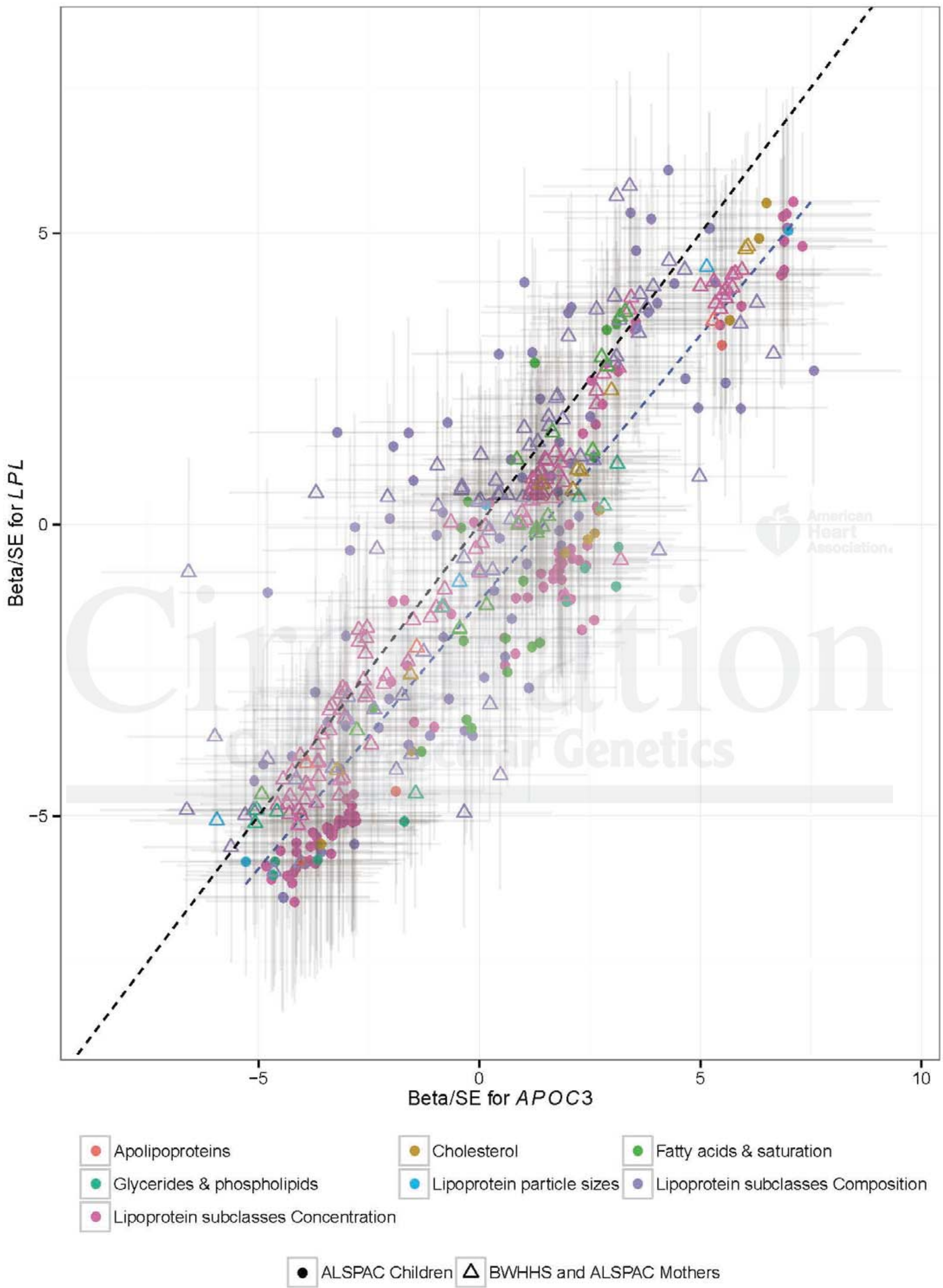

American Heart Association
Circulation Cardiovascular Genetics

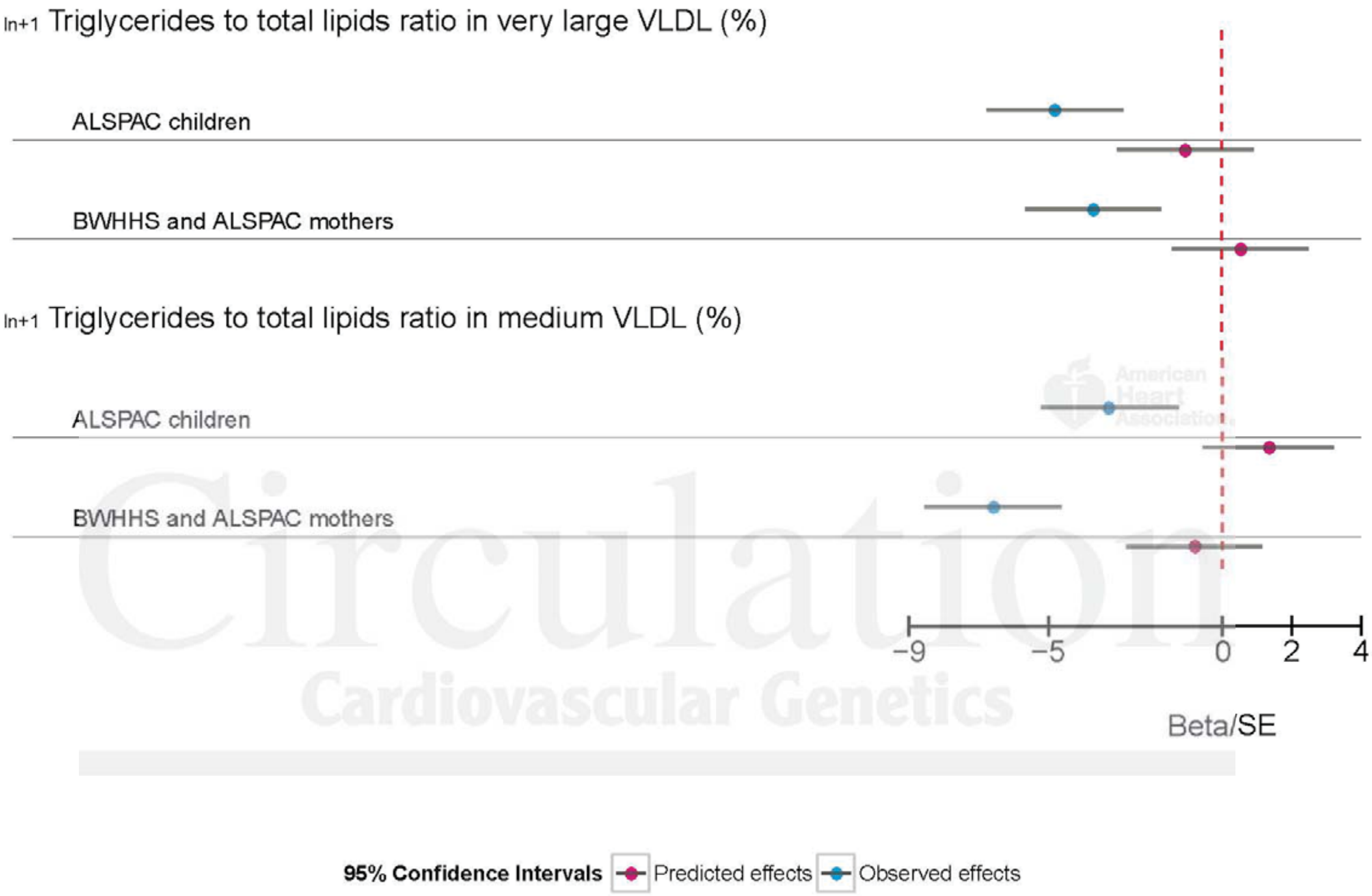

American Heart Association
Circulation
Cardiovascular Genetics
